# Supplementary material for: Molecular Characterization of an Isoflavone 2′-Hydroxylase Gene Revealed Positive Insights into Flavonoid Accumulation and Abiotic Stress Tolerance in Safflower
Source: Molecules. 2022 Nov 18;27(22):8001. doi: 10.3390/molecules27228001 (PMC9697648; doi:10.3390/molecules27228001)

(Figure S1)

Pairwise alignment result and the identification of conserved amino acid residues in safflower CtCYP81E proteins. The indication of he colored boxes represents 50% simillarity of amino acids.

(E)

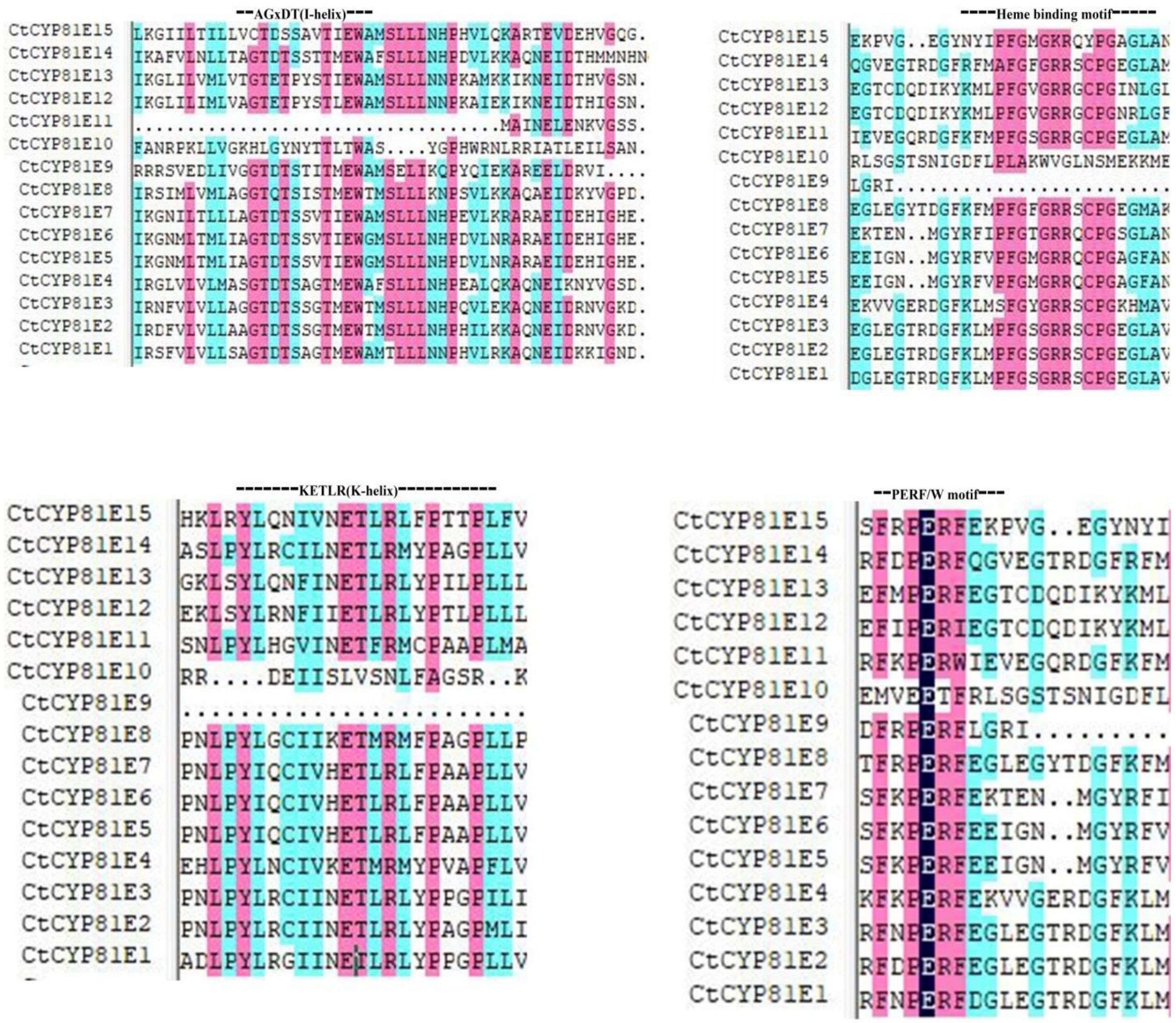

Supplement: Supplementary file 1 [file molecules-27-08001-s001.zip › molecules-2004143-supplementary Figure S1.pdf]
