# Supplementary material for: Molecular Characterization of an Isoflavone 2′-Hydroxylase Gene Revealed Positive Insights into Flavonoid Accumulation and Abiotic Stress Tolerance in Safflower
Source: Molecules. 2022 Nov 18;27(22):8001. doi: 10.3390/molecules27228001 (PMC9697648; doi:10.3390/molecules27228001)
Supplement: Supplementary file 1 [file molecules-27-08001-s001.zip › molecules-2004143-supplementary Table S2.pdf]

Protein sequences of CtCYP81E members identified from safflower genome.

>CtCYP81E1

MEVFLYLFISILLLLSSYLFTRRKIPNLPPSVFPSLPLIGHLYLLKPPLYRTFAKIASKSGPVLLLRL
GSRRVLLVSSPSAAEECLTTNDVVFANRPRMLFGKIIGMNYTSLAWSPYGDNWRNLRRIAAT
EILSIHRLNEFHDVRVDESRLLVRKLMIPFSIPSNSYSSSSSSCSHVNMKSAFYEFTLNVMMRMIS
GKRYFGGDIPAVEEEGRRFREILDETFLLAGAANVGDYLPVLGWLGVKGLEKKLIRLQEKRD
VFFQGLIEQLRKSKGGESENKSKRKTMIEVLLSLQESDPEYYTDAMIRSFVLVLLSAGTDTSAGT
MEWAMTLLLNNPHVLRKAQNEIDKKIGNDRLVDESDIADLPYLRGIINETLRLYPPGPLLVP
HESSDDCVVAGYNIPSGTMLLVNQWAIHHDPNLWTEPERFNPERFDGLEGTRDGFKLMPFG
SGRRSCPGEGLAVRVLGSTLGLLIQCFDWERTSDKMIDMTEAPGLTMPKAEPLVAKCQPRLE
MQNLLSQL

>CtCYP81E2

MEISYLYISFLLLLASYLFTFHFRRRISNLPPTVFPSLPLIGHLYLLKPPLYRTLAKLSSKHGPILHL
RFGSRRVILVSSPSLVEECLMKNDIVFANRPRMLFGKIIGQNYTTLVWSPYGDNWRNLRRIASI
EILSIHRLNEFHDIRVEEGRLLIRKLLSNSSPVNLKLVFYELTLNVMMRMISGKRYFGGDKLEEE
GKQFRAILNDAFMLAGAANMGDYLPILSWLRVNGLEKKLIALQERRNVFFQRLIDQLREGN
GNQAGNKRKTMIELLLSLQESDPEYYTDAMIRDFVLVLLAAGTDTSSGTMEWTMSLLLNHP
HILKKAQEIDRNVGKDRLVDESDIPNLPYLRCIINETLRLYPAGPMLIPHESSHDCVVGGYNIP
RGTMLLVNQWAIHHDPKLWSEPERFDPERFEGLEGTRDGFKLMPFGSGRRSCPGEGLAVRM
VGVTLGSIIQCFDWERMSDAMVDMSEGPGLNLPKAEPLVKCKPRLEVQNLLSQLM

>CtCYP81E3

MEISYLYISFLLLLASYLFTSHFRRRISNLPPTIFPSFPIIGHLYLLKPPLYRTLAKLSRKHGPILHLR
FGSRRVVLVSSPSAVEECFTKNDIVFANRPRMLFGKIIGLNYTTLAWSPYGDNWRNLRRIASIEI
LSIHRLNEFHDIRVEEGRLMIRKLLSGSDPVNVKLVFYELTLNVMMRMISGKRYFSGEKLEEEG
KEFREILNDTFILVGASNIGDYLPILSWLGVNRLEKKLIALQERRDVFFQRLIDQLRKGNGNQA
GNKRKTMIEVLLSLQESDPEYYTDAMIRNFVLVLLAGGTDTSSGTMEWTMSLLLNHPQVLEK
AQNEIDRNVGKDRLVDESDIPNLPYLRCIINETLRLYPPGPILIPHESSDDCVVGGYNVPRGTM
LLVNQWAIHHDPKLWSEPERFNPERFEGLEGTRDGFKLMPFGSGRRSCPGEGLAVRMVGVT
LGSIIQCFDWERMSEAMVDMSEGPGLTLPKAEPLVAKCKPRLEMQNLLSQLT

>CtCYP81E4

MTNGMMESISLLDYATRSFRMTTGEPDLGYFMPILKLLGLNGLEQRSKQLQKKGDLLMDNLI
EDLRTTMLEFSDGSGDRREEKVLEFLLARQKDDPKRYPDEIIRGLVLVLMASGTDTSAGTMEW
AFSLLLNHPEALQKAQNEIKNYVGSDRFLEQSDIEHLPYLNCIVKETMRMYPVAPFLVPHESS

KECKIGGYNVPKGTMLMVNVWAVQSDPNIWAEPTKFKPERFEKVVGERDGFKLMSFGYGR
RSCPGKHMAVRVISLALGSLIHFFDWERVSEKTVDLTEETGLALLKAQPLVAIGRVFEESAVKP
KNGSKEDVGEAWTSIEDGGKLKKSELF

>CtCYP81E5

MDKVYLFFISLLSLLSIILLFKPFTPRKRRNLAPSPPSLPVIGHLHLIGEPFHRLLHHLSSKYGPV
MSLRFGSRPVLVVTSPSAVEECFTTHDIVLANRPLLLSGEYLDYDHTTMGSAPYGRLWRDLRR
IATLELFSAARLKAYMGVRHDEARSLVKSLSQDVSHDFTRVEMKSRIEGLSYNIIMRIVADKRF
YGTEVDGFEEARKFKDLIREIFEVSGASNAGDFIPFLRWMDFQGLEKKLRKLQTESDSFIQRLIE
ERRSKRDSTSSCDGKAKTFIDAMLSLQESEPEYYTDDIIKGNMLTMLIAGTDTSSVTIEWGMSL
LLNHPDVLNRARAEIDEHIGHERLVEETDLPNLPYIQCIVHETLRLFPAAPLLVPHEPSEDCTI
GGFHVARGTIVLVNAWAIHRDPQLWDEPLSFKPERFEEIGNMGYRFVPFGMGRRQCPGAGF
ANRVVATVLASLIQCFEWKRVGDELVDLSEGKGLTMPKEKPLEAMCKVRQRMSRVLSGL

>CtCYP81E6

MDKVYLFFISLLSLLSIILLFKPFTPRKRRNLAPSPPSLPVIGHLHLIGEPFHRLLHHLSSKYGPV
MSLRFGSRPVLVVTSPSAVEECFTTHDIVLANRPLLLSGEYLDYDHTTMGSAPYGRLWRDLRR
IATLELFSAARLKAYMGVRHDEARSLVKSLSQDVSHDFTRVEMKSRIEGLSYNIIMRIVADKRF
YGTEVDGFEEARKFKDLIREIFEVSGASNAGDFIPFLRWMDFQGLEKKLRKLQTESDSFIQRLIE
ERRSKRDSTSSCDGKAKTFIDAMLSLQESEPEYYTDDIIKGNMLTMLIAGTDTSSVTIEWGMSL
LLNHPDVLNRARAEIDEHIGHERLVEETDLPNLPYIQCIVHETLRLFPAAPLLVPHEPSEDCTI
GGFHVARGTIVLVNAWAIHRDPQLWDEPLSFKPERFEEIGNMGYRFVPFGMGRRQCPGAGF
ANRVVATVLASLIQCFEWKRVGDELVDLSEGKGLTMPKEKPLEAMCKVRQRMSRVLSGL

>CtCYP81E7

MSLRFGSRQILVVTSPSAVEECFTTNDIVLANRPLLLSGKYLDYDHTTMGAAPYGRLWRDLRR
VATLELFSAARLKGYMAVRQDEVRSLVRSLLSHDACRGFTRVEVRSRIQGLSFNVIMRIVADK
RFYGAGVDDVQEARRFKDVMREVFEISVASNPGDFIPFLRWVDFQGLEKKLQKLQAKFDSFS
EALIEERRSKREGSSGESEAKTYIDALLSLQESEPEHYTDNIIKGNILTLLLAGTDTSSVTIEWAM
SLLLNHPEVLKRARAEIDEHIGHERLVEETDLPNLPYIQCIVHETLRLFPAAPLLVPHESSEDCT
IGGFHVSRGTMVLVNAWAIHRDPQLWDEPLSFKPERFEKTENMGYRFIPFGTGRRQCPGSGL
ANRVVTSALASLIQCFEWERVGEEMVELLEGKGLSMPKKKPLEAMCKARQRMSHLLKEL

>CtCYP81E8

MMRMISGKRYYYDVDDVLITVEEKEKADWFQELVTEIFEVMGATNVGDYLPMMRWLGVSK
LEKRMIRLQAKRDLFMQELVEELKRGLNKSTDDQEKKKKKKKRRNNMIDMLLSLQKTEPEF
YTDEMIRSIMLVMLAGGTQTSISTMEWTMSLLLKNPSVLKKAQAEIDKYVGPDHLIQESDIPN

LPYLGCIIKETMRMFPAGPLLPHESTKDCKVGGYHIPGGTMLLVNVWAIQNDPTIWGDPKTF
RPERFEGLEGYTDGFKFMPFGFGRRSCPGEGMAKRMVALGLGSLIQCFDWERTSESEVDLSEG
TGVSMPKAINLVANCVPRSTMLNLLSHL

>CtCYP81E9

MSSDGNIADASGRRRWSSTVVTDDGGRRRSVEDLIVGGTDTSTITMEWAMSELIKQPYQIEKA
REELDRVIDCKMAGYDITKGTIALVNAWSIGHDSDHWDDPEDFRPERFLGRI

>CtCYP81E10

TSSVNSPPRPFFSLPIIGHLYLLNQPLHRTLAKISNCYGPVVFLELGSRPVLLVSSPAAAEDCFTT
NDLTFANRPKLLVGKHLGYNYTTLTWASYGPHWRNLRRIATLEILSANRIQKFTGIRRDEIISL
VSNLFAGSRKDRFVVADMKSCFFKVTLNTIMMMISGRRCYGEDMEGPVAGKLQEMVEETFR
LSGSTSNIGDFLPLAKWVGLNSMEKKMEELNLKKDSAMQEMIEWHRRMRSDSACL

>CtCYP81E11

MAINELENKVGSSRLMNDSDLSNLPYLHGVINETFRMCPAAPLMAPHESTKECFVSGFRVPA
GTMLLVNLWAIQNDPKLWKEAERFKPERWIEVEGQRDGFKFMPFGSGRRGCPGEGLAMRM
VGLSLGMLLQCFEWERIGVEKVDMSEGFGLTMPKASPLVAKYQPRPHMVDLLSRI

>CtCYP81E12

MLLRFGARRVLVVTSPSAVEECFTKGNDIVFANRPFAAFGKYLNYDNTTMGFAPYGHHWRA
LRRVAASELLSTAHLDSTTTSREHEVKLCCREIVRESLSSQKIELRSRFSGLVNNITTMAMMGK
RYYGDGVEKGDERSAAIYREIMREMFKFIHTQNLGDFLPILGWVDFGGTKKKMVSLMKRVDE
FLDEVIEVRRRIGRCGSGIMVDKLLELQEEVPEIYTDQFIKGLILIMLVAGTETPYSTLEWAMSL
LLNNPKAIEKIKNEIDTHIGSNRLLQENDLEKLSYLRNFIIETLRLYPTLPLLLPHSASKDCKIGG
YLVPGGTMLIVNAWAIHRDPILWDKPEEFIPERIEGTCDQDIKYKMLPFGVGRRGCPGNRLGF
RVLGLTLGTLIQAFEWEKIGNEDVDMTAFYGTSMSKLKPLQALCKPRSMMAHFV

>CtCYP81E13

MLLRFGVQRVLVVTSPSAVEECFTKGNDIVFANRSLSALGKYLNYDYTTMGFAPYGHHWRA
LRRVAASELFSTAHLDSTTRSREHEVKLCCREIVRESLSSQKIELRSRFSGLLNNITTMAMMGK
RYYGDGIEKGDERSAVIYREIMGEMFKFIHTQNLDFLPILGWVDFGGTKKKMVSLMKRVDEF
LDEVIEVRRRVGRCGSGIMVDKLLELQEEVPEVYTHQFIKGLILVMLVTGTETPYSTIEWAMSL
LLNNPKAMKKIKNEIDTHVGSNRLLQENDLGKLSYLQNFINETLRLYPILPLLLPHRASKDCKI
GGYIVPSGTLLMVNAWAIHRDPILWDKPEEFMPERFEGTCDQDIKYKMLPFGVGRRGCPGIN
LGLRVLGLTLGTLIQAFEWEKIGNEDVDMIAFYGTSMSKLKPLQALCKPRPIMAHFSKV

>CtCYP81E14

MDYYLFLTLLLLIILLPFFLAAHFRRKTQNLPPTIFPTLPIIGHLHLLKTPIHRTLAAISAEHGPIL
LIRRVLLVSSPSAAEECFTKNDIVFANRPRLLAGKILGFNYTSIGWAPYGDHWRNLRRIAAIEIF
SSHRLNDFHDARADEGRLLIQKLVSDCSSPARANLCRDQIHHWSPVNLKSVFQELTLNVMM
RMISGKRYFNEEMDDEEGRRFREIVKETFVVGGASNLVDHLPVLSWLGVKRFEKKLVSLQKK
RDSFVQDLIDQFRNAKPKDINDNKKKTMIEVLLMLQETDPDYYTDELIKAFVLNLLTAGTDTS
STTMEWAFSLLLNHPDVLKKAQNEIDTHMMNHNQINRLVDESDMASLPYLRCILNETLRMY
PAGPLLVPHESSEDCVVGGYHIPRGTMLLVNQWAIHHDPALWSEPGRFDPERFQGVEGTRD
GFRFMAFGFGRRSCPGEGLAMRMVGLTLGLLIQCFDWERVSEEMVDMSEGAGLTMPKAQP
CVAKCRPRPMMHKLIS

>CtCYP81E15

MTIFTLQFGSYPVLILSSPSTVEECFTKNDVSEDRPRLITRKIMKYDYTSIIAAPFGPYWRKLRRIT
TIELFSITRLSTYLDIRQDEIRSLIKTLFRESVDHDYFTKVGLRSKLQDMSFNIIMRIVSGKRNFGP
ELNDLKEEINFRDMITEILKVGRGSYRSDLFPFLRWIDFQGMKMTLSRIKAKRDAFLDENGVK
DGVSNNKMIDAMLSLHESKPESYSDQILKGIILTILLVCTDSSAVTIEWAMSLLLNHPHVLQKA
RTEVDEHVGQGHMIQEHELHKLRYLQNIVNETLRLFPTTPLFVPHESSKDCTIGGYRIPRGTM
LLVNTWAIHYDPKVWDDPTSFRPERFEKPVGEGYNYIPFGMGKRQYPGAGLANREVGMAL
AAMVQCFEWDRVTEKMVDLNEGKGLIMPKNEPLEAMCRTRESMVNVLSKL
