# Supplementary material for: Molecular Characterization of an Isoflavone 2′-Hydroxylase Gene Revealed Positive Insights into Flavonoid Accumulation and Abiotic Stress Tolerance in Safflower
Source: Molecules. 2022 Nov 18;27(22):8001. doi: 10.3390/molecules27228001 (PMC9697648; doi:10.3390/molecules27228001)
Supplement: Supplementary file 1 [file molecules-27-08001-s001.zip › molecules-2004143-supplementary Table S4.pdf]

Promoter sequence of CtCYP81E encoding genes

>CtCYP81E1
attaaaagaagaaaaaccatacatattattattattatcaaaacagtttttgattgaatatgtaaaacaaaacttacaaaggtaatattgtaca
acttcgttttgatcgtgagaccataacgtttatgaattcgtgaaattgtggacgatgagattagataaatttataaaattgtaaaaattaaagt
taaatatgtaacaacacaaaaataacaaaataacaaaaatgaaaaactaaaaaaaatgaaaaaaagttcacgtcttacaaaaaacgtgc
gccgtggcgcgatatggcgcgccttgaggcgccatggcgtgagccacgccttatagaaatgctatgggacatttcgtatcggcagagac
gcgtcttacgccatattgcgccatgatgcacgccatgacacgtctttttgaacactggatataacacacgtattggataaaataaatggtag
aagtgagaaaatgacatggcgctattatagcattgggtaaggaaggataatttggttcgcattaggggtgttcagtatttgaatgctattcg
taaaaaccgaaattcgaaccgactcgaactcgaataatttaatacaaatcggtttgtttggttttccgactcgattcgaaagtttacatttggtt
ttcaaatcggttttcgactcgattcgaaagtttacattggttttcgaatcgaatttcgaatttataaaaatattcgaaacccgactcgaaattcg
aatatatatatacacacacacataagattatatatatacatatatataaatttcaagcttctgattgctagttatccacattgtgggaagttttctt
tgggtttttgttggttatctacactgcaggaggtttttttgggttatctggagtttgttatctgatatttttttgttgttatatggaattttggatgc
tttccattgttagttgttatttgatattttgtaatatggatatttgtatgtttgatgctcaaattgagtgaatgcctaaatgtaatgcggatgttcat
cctttttagatcaaattattagtttttatccccttattttgtagtagaatatttttgggggggggtggggggggggggggtagggtaggag
gtggtgtgtggctctcggtttggggaagttgaaccaaacgaacctgaaatcaacacggtggtgttctcgatctacccaatgaagccgacg
gccaagccgaatgcttcggctttaccatagcggatggcttgagcaatttcatcaaatatgttatgttaactttatcgtatatatccattatgcat
gactcatgtacttaaaaaaatgtttttaacccacactttttttattattttaatataataatatgagagagaggagagggagagagagttacgc
tcggggcttggaatgccgcccacggcccacctacccgctggccgcgtaccaccgacccaccactggccgaacccgccgccgatgtgaac
ccgtcccctacccacatgcacaatggatggcttaggtcatgcgtatccaaccctcccaaccctactaccagtgtctgccacatcagtttttctc
caaccctcccattaaccctctagaccctctctgattagtacccaaccctctcaaccctctcatttaattaaacacacccctcacacacaccacac
acacagcacaagccgctgtaagcggtgtggacaaccctctagcctctcaaacccgtcggtagcggaactcaccgaacccgctgagccca
gcaggttcaagccagccccgcggtacgcaccttcttactttaaaaaaatgtttttaactttttcccccctataaatcacaatattcttgacggacc
tctgacgtcacaagtcgtcgccaggttggtaaacgcccccataagtcaaccctcttcctcctctttcacgttatataaccaaaaaccaatttcc
gccattaaaacacacacacacaccaagctcaccttaaaaa
>CtCYP81E2
gcggttcgtatggttggggtgacattgggttcgatcatccaatgctttgattgggaaaggatgagtgaagccatggttgacatgagcgaa
ggtcctggactcaccttgcccaaggctgaacccttggtcgccaagtgcaaaccgcggctggaaatgcagaatctactgtcacaactaacg
tgatcagtctgttatcgaacctttatttcatttgaaaacaagttggcaatggtgcattttccacgggtgctagacgtttttgggtcgggattac
gaatatttgtatgataatatatgtgtatatgtattatgtatatgtatatatagatcgacctgatcgaatttgaaaaacttagatggctaagtatg
taaaaggatgttcatggagtggtatacttgtattgaaaagtgttcaaagaaagtcgctcaaggacccaatgacgaaggaggaaatcaaa
ttcgcggttcgacgattttccgatgactaattataaaataaataaaaaaccaccgaaaagcagtcgttaccggccatcgtaatacattgttttt
tttagtagtgaccatttccccagcatgggcgctttgagagagctttcaaactaccaattcccaatttcctttgtcctatccgattgagtaccttttt
cccacgcggcccaatacattttctatttgtcttccgtgcgttcccccaaaaaaaatcgacatggatactctcaagagcgcgaataactttgtta
ggagcctcgaaaagttagaagaaatatgtacccaaacttcctaacaccaattcgcaaaggcgcaatcattggtcgatttattggaaacccc
aaattggtcgaaatccgttactcatccgaaaaaaaattaccaaggattatgtccatcatcaatcaaggacaacatgtatttttgtgttaggaag
cactcaaccgtttagtctatctattggtaaaaatgtgatttctagtgaattcattatttcatccattttcaatgtaacaaaaaggggaattaatttt
gtctcattccttccctttgttctaatgtctagatcaatgaaacaatatctttttttctaattctactacgtcccaaacatagtctacaagaaaacaaa
taattaaatacataaagtggaaaactaaggatccattaaggctatgtttgacaaactagctaataagctagctatagctggtagctgataa
gctaccggttgaccgataagctagctagtaactgataagctagcttatggctgtgtttggcaaaaataagctagcttataagctagcttata
agctaatccatccaaacatgtaccagaagctagcttataagctagctttttgaaacgctactttgagtagcttatgagtagctttttgtagcttt
ttccaattatacccttattaatattaattattaaattcaaagataccctttttggtcattttacatactcataagctagtttttgccaaacataatttgt

tcataagctagcttatcagctactaacaagcttatcaatcaacagctagcttatcagctatcagctagcttatcagctaccagctagcttataa
gctagtccatccaaacatagcctatgaacaaattatgttcggcaaaaactagcttctgggtatgtaaaatgaccaaaaatgatatactagg
gtataataattaatattaacatgtgtataattggaaaaaagctacctccaattagcgtttcaaaaatgaccaaaaagctccaaaaagctacct
gcaattagcttttcagaaagctagcttataaagttagtttataagctactttttatccatcaaaacatttacacaaaaaataagctagctcactt
ataagctagtttgtgtttagcaaacaaaagcctaagtattaaatatttgaatttggttttcctataaaaccaagaacaatatccaccattaaac
atattacatacaccaattaattaagaacttcagca
>CtCYP81E3
tggacgagtttttgaggaaagtgcggtgaaaccgaagaatggatcaaaggaggacgtgggagaagcatggacgagcattgaagatg
gcggaaagttgaagaaatcggagcttttctgatgataaagcttgctgtccgcttaataagatctatcttgtaaggaagaaagccttcggtaa
aactgtaatgattaaatgtttttgtttgccttttaacggttccattaagagacaacctctaaattgttaagaggtatatcttgtttggcaaaaatg
aaatttgccttttaatggttctaaaaccctctgaatggttttgtatcctggaccccccttaaccattaagccatgggcggacctagcttagtgcc
tatggtggcacgtgtcaccgtttcattcctgcgtcatagtgtaaaaaattacacgactgtgaaaggtgtcaccgtattaatttattgtatgcca
ccatatagattttgaaactacaataaagtcacagatcttctttgatagagttggtaagttccttgcttttcaaacttaaggtcttgggttccagtc
ttgtttgagtcatttgtactaaattttagctttagtttcatatttattgtgtttataaaaaaacctgcttgcatcaagactcgaactagagactttac
tattcaaaagcaatgggcaaaccagtaattgctagaccaatataaattttatgaaaagtatttctacatgtatttcttatagtcaaattgtgcca
ccgtttgaataaaattctaggtctgcccctgcattaagcgtcctgatttcattctcaaccattaccaaacaaccatatttaattttacactatacca
taataggagccttttaatcattctgacaacttaatcatccatcactaattcattcagcaactgtcaaacaaccctcgtttgtatcattgctaaaga
tcaatttgtgtgtgtattgttaaattggattagtaagaatatatctaaatctctgtttaggtatgttaggtgagagttaggataaaatacatgat
gtaatgtcacttgtttcaatatttatataatacaacccctttccagttttaattaacatctgagtaaatgtggattaggtaaaactcttcatcatctt
cttctaataattttccaatcccttcagcgacaaaaaaagcgtcgctaatgctgatatccatcgttgaaccaccgctgaaagtccgtcggata
actgtagtgggtgatgctatagcgacgggtatcaacgatgatgagattctcctctgactgaactcaaatgttcacatataggtgttcaagaa
ggtcatatatcgtcatcgaatagagatcttatttcctcatcttcttgttgacgagaagctatacatcacaagtaaccgttagaaccaaccattta
caaattaaaaattaaaatgaattaagtttagtcaccggacggtgtaattgtggtgggtgaggtaatcgttccatgatatattcaaataacca
acagattttcgtggacctaaaaaagttttataagaataaggtcttgtaactataaccaacaaatcatcagtggatctattgtaattctaatccg
ttacttatccgagacaaattaccgagggttttctctatcgaggacagaggacaatccgtttttgggttaggaaattgtcgacggcttggtcat
ccgtcggtaaaaatgtgatttctagtagtgaattcctccttatttcattcattttcattgcaacaaaagggaaattatttttgtggcactccatccc
tttcttctaaagtcttggtcaactaaacaagtttttattcctaattcagttacctccaaaacttagcctacaagaaaattaataataaaatccata
aaatggaaaactaattaagcatgaagtcttaaacatttgaatttgattccctataaaactaaaaccaaaatccaccattaaacatacaccaaa
ttaagaacttctaaca
>CtCYP81E4
aacttggctattatttaatccaattcatactggctcctaattttgctttatatatgatgctaaatcatgtagtctataacattagttatttgctagta
acatgatatagcacttggtagtggtaactgcatgtttatgatataatttgttagtaatttcaattgattagttcatgatagatcaatgttagtgac
ttagtttgtcctctgggagtggttgtcatcttaggagagtaggattgccggcaaggaaagaaagcatcatgtttctagctacgatgtgtctg
cgagtctatcagcactggtttattaataactggtgatgtgttctatcaacgttgtgaactaacatgagactaagaatttttttattgattgtccca
gagactataaaattcttcagtcgatcattcgttgtgggtttagtagctttggggctcatttgtcccactaaaagttgcgtatgagttagtgacc
aagttcagttatcctttgaagaaggtcatctgccaataatcagcaacttcattccaaggaaactatcatcttcttcattgttgtattacatggttc
tctgtgcacacatgaatattagtttatatatatgaaactgagctgtttagatttaattttaaattaatttgattactgttaacatgtgataaagtac
tctaaaacatcggcctgtgtcaagtgtgcaggagtgggctggtagagtgtcggtgtagacagttgtttattaagttcgggatgtcgtacat
cggcagaagggtcctcacaactcacaggtgaaaatgggttccaactctggttgctcgtaatctgttacggtatttctttttctcttgtaggttg
cgtcaatttgctgtcgtcatgtgtaccctactgcattactttttgtcattttgggtgtactcttagttgaggtttgttagaaggtgctgctgtgtttt
tgatcgagaggcttgtattgtggtctgttggtatcttctttgtactttaaaacccttatgggtttctttttgttggtaatataatgttctaaaaaaa
gattggatgctttcttattgaaggcaatattggatatcaattttagtttcttttggagtgcgtttgatgctcgtatttgtattcaatgaaattgcat
gattacatttaattgagttgcataattacacaactagtatatatatatatatagagttataacggtccgttacacaccgttatatatatatatatt

gttgtataggaaaacaaaaaaatatttttaaaaatcaaaattttactgacgaattagtgatagattttctgttggtaattaaaaattattattta
tttattttttagccgtcggtaatccgtcggtaaaataattatttattaaattaaaaaaaatagataataaataaattccgtcggaaaaccgtcg
gaaaaattaaataaaaataattatttattaaattattttaattagaaaaaattaatttccgtcggaaatccgtcggtaaataaaataaaaataa
taatttactaaatttaatttccgtcggaaatccgtcggaaattaaggccgtcggtaatccatcggtaatttccgtcggttatccgtcggtaatg
agattaacgaccagcaaaataccgacggaccatttccgacggatttctgaccgatttaccgtttttccgacggatttccgacggaagctggc
tgtcggaaatacctttttttcttgtagtacagtgttatttatatcattacaattcatagtttaactttaaggattctagaaagtaatgatttttggag
agtttctccaaatatggagattcccatcacctctttccctggtttctccattttgtagaaggttttggagaatatgttggagcataaacctacaa
aatcttcattttggagaatctgctaaattaaatgtatatacaagatttttctcaatttttagtgtttgtaatatataggtattgccacaatacg
>CtCYP81E5
cacatcagttttctctatcttatacatttcataaacctttcactctcaatcatggacctactcatttgttattacctaaagttatgttaattaataaaa
aaagcataaaaacactaagttttgaatttatttagtattaatattgagtgtggggtccatgtgtaagaaaaagaaaaggttcatacagatga
acctttcccatgccacctaagcattaaaaggtccttcccaatggttgggaattccatggaaaggtccatggacctctccattctcaccattgg
agatgctctaaggggtccatatataattccccttttgatatagggaattttaaatggacctgagaggtaatgaaaggtttgtatatgtactag
gactgaatataacaggatctacttttacgtgaatgataaaacatctgtaaaggatccacataacatcaccgtttgctatggataaccatactg
caagttacaagcattaatgtccttattcggcagtcaaaagaggtggcgtattattttattgatgcatatatatatatatatatgtgtgtagagg
aaagttcaaatgtgaaccacttttattgctactgtgtagagcaacagtatccccacgtctatgccgtgatgccctaacatttgtgtcaactattt
gcaataaggctaaaaatttaatctatcttttcttgtttcatttttcgttatatatatatataggaaagttcaaatatgaaccactatttggttgtaaa
ggaccactaaaatcgtaccacgtggcaatttgaatcggtgcatacgcttcattcagtatgaacattatgcaggcgcgcctaagctttgttca
gactgagtaaagcttattcagactgaataaaacttcattcagtctgaataatacatgaatattcaaactaaatattgcacgtggcgcgatttta
gtggtcatcatgttcacaaccaaaaagtggttcacatttgaactcacccctatannnnnnnnnnnnnnnnnnnnnnnnnnnn
nnnnnnnnnnnnnnnnnnnnnnnnnnnnnnnnnnnnnnnnnnnnnnnnnnnnnnnnnn
nnnnnnnnnnnnnnnnnnnnnnnnnnnnnnnnnnnnnnnnnnnnnnnnnnnnnngctataa
aattttaaggctaaattgataaaagaataagctagcttattgaaaagctagttggaatagtgttttaggagtttttttgtagatttttttcaattat
acccattattaatattagttattataccccaagatactatttttgttcattttacatactaataagctagtttttaccaaacataatttatttataagct
actttatcaactactagcttagcttattagtcaacttatcaaccatcagttaacttatctcttagtttgccaaacccatgttaattaaatatttataa
tgaataacgtgataaaatatttaaaattgaatagttatcaataaatggttcttgatatcttaaaataacctaattaagaggtgatggaaaaac
tataaaaaatatataaaagctgatgaatacaagaatgcatctttaactgtatccatatttatatatatatggccggccttgatatcttaaaataa
ccaacttaggaggtgacagaaaaaactataaaaaaataaaagctgatcaacattctcacacgttaaactcaacttttcatgatccttcacata
tatatagaggatgataaccaacttttcaggatcaaaccagaatactatctacttaaaagaacatgataaccaataaaattacataaaagtcg
tttccaaagtcgtccgcttggcttttcggaaagttaatacatatgattttattggttttcgtgtttttttcagatatatacttgaagatgactctctctt
tatatgtatatatatacctgtgtgtgtttgtccatatacatcacaacaaccccctcttcatc
>CtCYP81E6
aacggtttgttaaaaaaccattacaaaaagtcacataaacatttagaaagtagttatggtaacggtttttataaaaaatcgttaccactacca
aaaacctagaaacataaagtaatgatttacacaaaagcgttactaaaaagtctcatacggtaatggttttgaaaaccgtcatttcatgttgtg
acggtttttaaaaaccgttgtcataaatgaccatttagtaacgggtttctctaaaccgttgttctatgttgtaacggttttaaaataaccattgcc
aaaactatggtataaatcgcaacgtttatttgaaaaccgtttccataaatcactaggttctatggtaacgattttaaaattttaagtcaccaaaa
aatagtttatagtaacgattttataaaacttatgtcaccgaatggcattcaaaggtaacggtttaaactacatttatgtcaccatatgttatgtta
cggtgacgattttgaaaattttacgtcactatatgtcattctatagtaacggttttgattaccatttgtaatgctacagtaatggttttaaaaata
aatgtcattgtacgttatattatggtgataattctaaataataaacgttgttatgtgtcatgctacgacgatggttttgtataagaaatgtcacc
atgtcatgtcatgatgacaacggtttttttattataaacatcattttactttgtgatagagtgattattttgaaaaataaacatcactatagaccat
cctacggtgatggttatgatatataaaagttgtcttatgccatgatacagtaatggtttttaagataaatgtccccttatgtcatgttacaatga
tggttttataaaacaactgtcactgtacatcatgctataatgatggttttggaaaaataagcgtcactgtatgtcattctaaaatgacggttttg
tcgagacgattataatggtataatttgttgaaaagaaaaaagaaaattttaatacattttagaataaaatgctaacatgacgattttcagaat

aatattgtaacacccagacaaatctcatatcgtcgtggacaggagtgatgatgggttcataagtaatattttggcatttctaacaacaacgc
gtattggggccacatgcacggcaggttcttggaacgctgcagttaagcgtgcttatgtgggagtagtaccaggatgggtgaccccctgg
gaagtcctcacttggtctccagaaacaaagctgtgagccagtggagcaaagcgtacaatattgtgtcgagtggggcgagttgttacaatg
tggtattaaagccgctcctgtgtcgttcgggatatagggcaaacctcatcgatatggtaacagtttgaaatacaaacgcgaccgtatgttat
actaccgtgacaatgttcaaaaataaacgtcaccataattcaagctacactgacgcttttgaaaataaacgtcatcatatgtcatgctttagtg
acgattttcaaacacaaacatcaccataagttatgttgcgatgacggtttgaaaaataaatgtcaccatatgtcatcttatggtgacagtttta
ggaaatattttactttttttacatatcaaaggtgacggttttaatcaatggtaacagtttttgatgtgatttggtaactttttttaaacgtaacgaa
aggtatatcaaaagtgacggcaaaatagatgacgtttttttatgttcacgaaaaccgttatcgtatacctatagtaacggtttttaaccgtcac
aaataaccattttgtactagtgaataaaaggaaacccaatgacaaagaatttggaatgtatagactcgtgaaatgcatgggatggtttca
cgatttgaaaaatgggaatggaattgtggaatgcatgggatggcttcattttcattgtttggcaaactggaatggaatcgtgaaatgcatg
gggatggtccaccatttaggattttaggccgcct
>CtCYP81E7
atttgcttttcagaacatcttgaatcacttaaatcgaggatttagtttgtgagatacgattaaaaccataggcaaatctgttggcatccttctttt
gtttctttattttggttccgtgattcgatcaacaatgtttcattttcaattgacgtatttaatttgaagtttttaagaaataaatacacaattatata
tatatatatatatatatatatattattcagactgtcagtttacagaaacagacaattttatttattgattcagattttaagcttgaaacatcacc
acttaaaaattagaagtgcaatcgaacttcagacttcaacaactcctgaaaaacttacgaagaaaattaggttaattataggattaatttgtc
ttcgttgtactataaatataaatgaccttaaaagaaggtttttcttttgtatttaaaagcttgaaaaacttttctaaaagtgtttttagataaaact
tttactttcattcaacaaccttccctatatataactgtagcaaaatgttgtagggaaggttttaagatggttttagttaggaaacatttatattta
gttgcacttttcacatttgttatataattcttttctctaaaagccactttttagatacgtttccaaatccttctctaaaactttctttttaaaacccctttt
tcttgtagtgttatcaaaattgatttttaagaatttaaacatgatttttttgaacaattaaagaaactgacaagttaaaagatggaaaatcgaa
gaccgaaaaccaaaaaccgaaccattttaaaccatttggttttaaaccgaaccgcaccgaaaaaccgaaaaccaaacgatttaaatgtcta
agaaccgaaggactcaattttaagaaagttgttctatccatgcactgttgcacaaagactttacaagaaattgtcgccccacatctataccg
tgatgcccaaacattaattgcccaccaacacaacgacttccattcacatacaaaacatcctatatatatatattatttatatatattatatatata
ggggtgggttcaaatgttgaaccatttttttggttgtgaacgtgaggacagccgagagcacaacacttgacggcattaagtgtggtccaac
cccttaattcaatgccctccatgaatcgcgcctagtcatccgtcgtaggcctgacatggcgcctagtcatccgccacgtgccgcgctctcgg
cttgtcctcacgttgacaaccaaaaagtggttcacatttgagctttcctctctctcttctctatatatatatgtacatgtgtgtgtatatgtatgta
gttcacatattgcaagcattcctgatcatggatgaaatccatctttctttcattttctcttttatccttcttctcttcatcatcttgctttccaagtttctt
atacccaaaaagacaagaaatcttgcaccatctccgccttctctcccattaatcggccatctccatctaatcggagaaccatttttttggttgtga
acgtgaggacagccgagagcacaacacttgacggcattaagtgtggtccaaccccttaattcaatgccctccatgaatcgcgcctagtcat
ccgtcgtaggcctgacatggcgcctagtcatccgccacgtgccgcgctctcggctgtcctcacgttgacaaccaaaaagtggttcacatttg
agctttcctctctctctctctatatatatatgtacatgtgtgtgtatatgtatgtagttcacatattgcaagctgatcctgatcatggatgaaatcc
atctttctttcatttctcttttatccttcttctctctcatcatcttgctttccaagtttcttatacccaaaaagacaagaaatcttgcaccatctccgcct
tctctcccattaatcggccatctccatctaatcggagaaccttttcatagactccttcaccaactctcttccaagtatggtccagtt
>CtCYP81E8
tgttaagcagtggtgcctgccgaatgagaggttgagggagagatctgggtttgcattttctgtccgtgaagggaagggggaagggtatt
ttgggaagacatgtggctaattagtggatacaacaaattgatggaagcttagcgaataagccattgttcaaatcaataagctgctaacaaa
catccatgtatggcatagcgaataagctatacatgttattaagctcattaagttacaaccaaacaggccgttagactaaagtggcgtgttcg
actatataatcgctaatcctttcagatggtaggcgtaaaaggagtaggcacaacttgttgagtaaagtttatcttcttattcttgtctcaggttg
atgtaatttctagcctctcgctagcttttccgtcttattgaattgcggtttcaaacaaaacttaatgcaattgaattttgtttttccttatttattttttt
cttaatttaaattcttgtttctaatatccatacatatattccaacttttagcctataaatagacacttttgcttccttagagattcatcccataataaa
ctttcattattgactttctctctattagagttttatcttgctattcatcctaataaaatactgtactaaaaatggaagcatttacttcttcatcccatt
aatcttcttagccatttccaccatctccagttacatcttccacaaactccagaaccacccaccaagcccatggctcccgccgctgcccgttatc
ggccacctctacctcctcaaaccccccattcgtccacaggtcctcgccaaaacggtccgatccaactcctgcaatttggctcacgtccggtcctt

cacatctcctctccgtccctagctgaagaatgtctaatcaccaacgacatcgtattcgccaaccgcccacggctgttggccgggaagtatctt
ggctacaattacacaagcctcgtctttgcaccctacggtgaccactggcggaacctccgccgtatctctgccctggaaatcctcctcgactca
ccggctcaacgagcttgcatccatgcgagtcgatgaagtgcgtctcaccgtccgtaaactctatcgggcgtcactgagcacgcggcggtg
gtgccggttagttaaagctatatttgttcactacaaaaaaaaaattgaccttcaaaagaatctttcttttgtatataaaaggcctaaaaacatcc
atcactaaaggttttcactcctcttaaaaagcttttctatataatgggtagctaaaaaatttaggggagggttttctagtagtgcattatgtaaa
tgtttacagaaagctcttttagcccatgcgtaccacaagatcgggtaggaggattcaagcgggttcggctggggctttcaagccctattaa
accgagcctctcccttccctctttctctctaacatatatattaaaataatatataaaaaatgagagggtatgggagtatgaaatgttactggta
ctccactgtccgattcagtgggtacgtaggaagagaaaatctaatgtggcactgagatagcatgagggtatgaggttatgaaactgttgg
tggtactcatgatcttaggctttagacacactttcaaagcttttctttaactttcagagaagatttttttttggcttgagatacccttcaaagcttt
ttcaagaggtggtggtggtgcagattaattatggctatgtccactacacttttcgctttcgaaaaaggttttcttgtgtatttaaaagtttgaaa
aaactttgctaataaaggttttaaagaaactttcacttccatcaaaaaaatttcccagttaaaagtacgttttaggcagggttttcaacagct
ggtgcatctatgttaacctaagcagctttttcggactttagatacgctttcaaagcctttctaacacacggcagtgcttcaggttaaagatatg
ttggttgacctttcgctgaacacc
>CtCYP81E9
tgttaagcagtggtgcctgccgaatgagaggttgagggagagatctgggtttgcattttctgtccgtgaagggaaggggggaagggtatt
ttgggaagacatgtggctaattagtggatacaacaaattgatggaagcttagcgaataagccattgttcaaatcaataagctgctaacaaa
catccatgtatggcatagcgaataagctatacatgttattaagctcattaagttacaaccaaacaggccgttagactaaagtggcgtgttcg
actatataatcgctaatccttcagatggtaggcgtaaaaggagtaggcacaacttgttgagtaaagtttatcttcttattcttgtctcaggttg
atgtaatttctagcctctcgctagcttttccgtcttattgaattgcggtttcaaacaaaacttaatgcaattgaattttgtttttccttatttattttttt
cttaatttaaattcttgtttctaatatccatacatatattccaacttttagcctataaatagacacttttgcttccttagagattcatcccataataaa
ctttcattattgactttctctctattagagttttatcttgctattcatcctaataaaatactgtactaaaaatggaagcatttttacttcttcatcccatt
aatcttcttagccatttccaccatctccagttacatcttccacaaactccagaaccacccaccaagcccatggctcccgccgctgcccgttatc
ggccacctctacctcctcaaaccccccattcgtccacaggtcctcgccaaaacggtccgatccaactcctgcaatttggctcacgtccggtcctt
cacatctcctctccgtccctagctgaagaatgtctaatcaccaacgacatcgtattcgccaaccgcccacggctgttggccgggaagtatctt
ggctacaattacacaagcctcgtctttgcaccctacggtgaccactggcggaacctccgccgtatctctgccctggaaatcctcctcgactca
ccggctcaacgagcttgcatccatgcgagtcgatgaagtgcgtctcaccgtccgtaaactctatcgggcgtcactgagcacgcggcggtg
gtgccggttagttaaagctatatttgttcactacaaaaaaaaaattgaccttcaaaagaatctttcttttgtatataaaaggcctaaaaacatcc
atcactaaaggttttcactcctcttaaaaagcttttctatataatgggtagctaaaaaatttaggggagggttttctagtagtgcattatgtaaa
tgtttacagaaagctcttttagcccatgcgtaccacaagatcgggtaggaggattcaagcgggttcggctggggctttcaagccctattaa
accgagcctctcccttccctctttctctctaacatatatattaaaataatatataaaaaatgagagggtatgggagtatgaaatgttactggta
ctccactgtccgattcagtgggtacgtaggaagagaaaatctaatgtggcactgagatagcatgagggtatgaggttatgaaactgttgg
tggtactcatgatcttaggctttagacacactttcaaagcttttctttaactttcagagaagatttttttttggcttgagatacccttcaaagcttt
ttcaagaggtggtggtggtgcagattaattatggctatgtccactacacttttcgctttcgaaaaaggttttcttgtgtatttaaaagtttgaaa
aaactttgctaataaaggttttaaagaaactttcacttccatcaaaaaaatttcccagttaaaagtacgttttaggcagggttttcaacagct
ggtgcatctatgttaacctaagcagctttttcggactttagatacgctttcaaagcctttctaacacacggcagtgcttcaggttaaagatatg
ttggttgacctttcgctgaacacc
>CtCYP81E10
taagggtgtggattggtttacacgatttagaccatttttgggttcacaaatgatctcccctcccctctctctctctctcagtatatatatatata
gagagagagaggagagataaggttcaaatgagaagtgctaaaaaatgagaagtagagaagtgatcaccactcatccggtcaataaaa
gcggctttgaccggttacggtggacgcgatcatggaggattgacgcgattcatgggagggtagagattattaaagaggggggaccacg
gtcaaagccacctctattgaggggatgagtggttgagatcacttctctacttctcatttttttagcacttctcatttgaacctaatactatatatat
atatatatagaaaggttcaaatgagaaacataacttttaagaaacgtgagcagcaatctcaaccactcatccggtcaataaaggcggcttt
gaccatagtcccccccctttaataatctctacccttccatgaatcgcgtcaattctccgtgatcacgtccaccgtaaccgaagatatgtggct

gagattgctcctcgatccctttctcataaattgacatgcttctcgtatagactccctataatatatatatacaatatacatatgtgtgcacgtatat
aaaaaggtgctaaaagaaattgtggttattttctaaattcgtgaaatctgataacgtcggaaatgtaccaaaaatcggggttagatgtagta
attgcaaccttacaaatactgtcctagccgtccatgacacctaatttatggtgcaagaatacaatagctgttaaaataatcaccttgaaaatg
aaagcttattagaacaccagttgaaaatgatagggtacgaaataaatagacttgaaaatgaaatatctcattacaaaattcagatagccga
ctaagagatgttaacaacagtgatcttatactcatctgtattcttgttttgttgaacacatgtgtcctttatacaaataatatcgagtgctgatac
aaaattaattgtttcaataaagtgttttacaagacttaaacttatgaaactaaagacttacaaataatatgtttataggatactttaacgtcttaa
ttattaacatatttagacgagtttaaagtaaaatatcacttattttttatatgatcatattaattgaaaaaaattagaacaacttaataagtgtct
tttaaaaaagagtaaattacatatttggtccctgtggtttacacattattgcactattggtcctattttaaaataattatgaaaatggtccctatg
gtttacactatattgcactattggtcctatggtttacactatattgcactattggtccttgtgatttacactatattgcgctattggtccctatgttta
acaaattgatttcacatggagttagggtttggggaccaatagtgcaatagaatgtaaaccatagggaccaaatccataattattttaaaata
gggaccaatagtgcaattaagtgtaaaccacagggaccaaatatgtaatttactctttaaaaaaatatcctcataaaacatttacccaattaa
aatgtttaattagagtaaatgtttaaagcaagtttcctaaagagcttatagagatcaacttatatatcacacaattgcttggttgcaaacctgt
ctttaatttgttgtgacaaaagagagcacgtcatgttatataacactcacgagatatttatcataactggtataaaaaatttcacgtttattgttc
aagattaaaatttaaaagaacaggtcccgtttctcccccgatttgtaacgaatttccctatatatacacacaataagattagtgcacttgataa
attaaccatctttcaattctagaccataaagatttctttcaattccatggagaagaacaacttgtactacatcctcataacttgtctcttcttatac
atgatcaccaaacatttccttc
>CtCYP81E11
tataatatttatatatattatagtattaggtttataagaaaagtgctaaaaaaaataccaagtagagaagtgatctctatcactcatccggtc
aataaaggcggctttgaccggtcaccctcgttgaatcaactttttgtaaaatgacgatcatgctctttatacccattttcagatcaaaggttta
gattggtcctcaagtttttacaaattatttggttttctcttgaaggcccctctatatattaatattaatatacataaatatgtatattaagtggaata
cacatatatagtaatatatataatatacttttacatataacctataaaacaaactaaaataaagcaaaaaaacaagaacggtaattctgtcatt
ttgcatcattgaaatcaaattcctttgacatgtctgcaaaccaaacagaataaggaattggattccaatttcatttcctgccaactaaacagaa
tatggaattggattccaattcctgatgaattccaattccatcaaaaacattccagcgaaccaaacgccaccctattacatcactcttacatatat
aggatacatatatatttggtgtcgttatgacattaactccaataaagttctttattaagataacatgcactacaaaacaaatgatttttacctata
aaaatcgtccaaggaaatttctatcacaaaataccaatgtttctagtagaatccgcgatatttttccacaaatcactcacaaattacaatgaga
tccctcacaatatgtcatttattcgagtttcccaccaacttttgtgacggttttatgatgaatcaccattttagcgacgaattactcaggaaaaat
tcagtaggtaaaagtcttccatttaatagtgatagagatttgtaatgtcatataaatcaaattttattgtagtgataacttttctttaaaattagat
tagaatcgcacaacttaatccaacatgattacttgttagatacaacaaaatcaggtttttaaaacgtgattatgtcaagatgttcttattaatgg
aatctttttatgggacttggatttgtaaaatcaaacaattatgtatcttatacaaatgctacaatataatttttgacctacttaggctaacatgtt
ttctaaaatacttttgaaaatggattatgaatttatttctctctttgatgcaaacataaacatctgcatcttctttgttaaaatggattatgaattta
ttttcatattagattatgagtttatttttgtatccaccatagttaagatgccttgttttctttctttttttctgttttattatcaattaccattgcttctttt
tttgttttgttagttgattctttgtttgaatgtaaaattttgatgcttaaaagtagtaatggttatgtgttgttggtttgttgcttatatgatactatg
aggtaaattggtctttttttacatttagacctttgattaagataaaaaggtaaacatcagcaccaatttattcatacagacaaatttatccataaa
ggacttaatgaaaattttggtaaacagccccttagtttttataggacatttttcagatatatacttccgatgtttataatttatatagtacagttatt
tttataacagattaattttcaaatattttcatataaaaattaattaaacaaagatgctttgttccaaaaatgttctaaaaaacttgttagaacactc
aagaaatattctatcaggttatttttaaataagtagtattctaacaacttactataatatcctgacatatttgacatcttataaatcctattcgttgt
agtgttcctaattaagatcagatcacaattaacaaataggtctatgttaacccatcttgtcggccttttatgttcaggttatgatatcagccgga
accgatacgtcagcaggaacactagaatgggctttatcacttctactaaaccaccccgattccctcaaa
>CtCYP81E12
atcagctttccactatactacccaaaactacccaattcatccactgtcgacccactgcaacccactacataaacttttttttaattaactaaattatt
tttaagtaattattttttaataaaataattagttaactttaataaaaataaataataatacttaaaataataataccatcgacgtcggccaaact
ttcttgagtatctcggggttcgtgtatatttggcatcggtataggagtagaatgagacaccatgggattcgattgagagagcattcccgcat
acaacggtagaagattggaaaaagaaacgttttggtttagcggtacgttggtgttaaaaaaattaggatcgtatacggaggcggatggc

gggatgacccaatcggtattgcgtggtatatatgaaccggtgggactcggcatttgtggtatatatgaaccggtgggagacaaatttgtc
ggcgggacttgggtttgtcggctcgaagtttttgattgtttgggagtgttttgtttatccatttggaggttatagtagaaagaaaaaagattg
aataagtagaagagaagttgtttggttgtgtgaaaatgaagtgtgaaagtttgggttttatagaagagaggttgtttttgaaattggaaaa
aattaaaaaaaaaaacaaaaaaaaagaaaaacaaaatatagccgttgggggggtgggggggggggtggcagctttttatttgtcagtat
ggggataccggtggcccaaggtaccggccgcctaccggcatttacaatgagagttcttcttaaggtttttacaagaatttctactgtgacag
agattcttatccaatcacaagttcttattagaacccaggttcttataatacttttcttttatgttatataataatttacataaataaaaattatgttat
agataagaaaaggaaggtgaggtttttgtaagcacctccattgtagagttttaagaaggttcttacattattaaggtggcaataatatgaca
taaaaaatatggcaagtaaggttcttaaaggtttcatcactatggatactctaagagagtcactttaacaaataaaaggatattgaaaaaat
aagcctattttgcaaattactctacaaaagatcaaaataagttctttactgaatgttttgtaattttataatattgtaaaatattataaacaccatc
ttagagctttttcaataatgatgttttaagatctttttaagaccatacattttatacatttatctatatttatctaaaagatataaatttcgatacaag
tattgaccttaaattttatacatctgacatgtaatttatagatacataaaaccaataaaattggcattatatgatggagaataaattgaacatg
aaacaaaattattctttcaagttctctttacacacgagattctttacgtcacatcatatgcgggtacgtatctaatatgtctacatctctacttgcc
ctagtacctctccaataatgcacaaaatgatggataaaaatctgctgtcctaaatttatctatttcgttcctatccaaccaataaaatgatgaca
tatcagattttattatgttttaaatttattttattcaccttcatattctatttctgaggatgtatctatcgtctgattggtgaacatgattggaatatg
aaaatttgggataggtgattttaatcgcaaggaacaacttcataaaattctctatatatatcttggtttgcctatgaagactacttggatcatgc
acaaaatgataggaattactttgttcaacttaattctcttgctaatatgcactattctcctattcatcttcaagctctccacaaaagcctctaaatc
aaattcaaacctacctccaagtcctccttcacttcccatacttggccacctcctcttactcaaacaacccctccatgaaaccctacacaccctctc
tcgaaaattcggcccaatc
>CtCYP81E13
attctcaaaggtcgtgaggcgtggcgttgcggcgaggccaagcctcaagccttaggagccatggcgaagacatgacgtttttaaggcgt
gacttatggcgtcaaattttagttcatataaaaatattatatgtaagatcatatgttttgtactgtcgttcaatgagttaataagaataagcaa
gttagaattaagttatatactaaaaactagcaaataaaaccttaaaaatgtaaagaaaaatcaaatatcgcattagaagaactgccagtag
accatggcgtgccatgggcttaacatggcgtgagacccaagccttgaagccatggcgcgccatggcgactgcaaggcgagtttttttaga
acagtgatatatatagaggttaaatcaaataaaaaccattttattagtgaaaaccagtgagaaccaactaaaaaataatttttttttacgatttt
tttccatcaaatgttagaattgatataatgtttcacatatatttttttttcataaggtgagtatcatcaatgaattggtttcataatatgaatcaaaat
tttgtatattttcgattcataatatgaataaaaatttatttttttgtatggatgatacttacattatgaaaaaaaatgtatgtgaaataatagttaa
aatcatagcatctgataaaaaaaaatcgtaaaaaaaattaagtttcaatcggttttacgggttttcactaataaaatggtttttatttgattaaca
ctatatatatatatatatgtagacttaggatcaggtgagaccaagctgtttccgtgagaccgcgtgaaaccaatacctaacgtataaaacttc
acaaaaaaacaccgtgaaggtaaataaaacacgaatacgcagacctaaaaatcactttcgatacaaaaaaatatccaaaacaaggtaaa
aaacgaaacattcacagctcaaatgctgtgaaggttacaaataaataatgcgaaccttcacagcgaaaatgctgttatggtttcaattttttta
cctttttttggattttttttcttcgatagtgtttttttagtatattttttacaatgaatgctcaattttttttacattcacaaatgttttgatgtgaagttttat
gtaaaatatgttggtctcaccggtctcacggaaacaacttggtctcaccagaacttcaccatatatatatatatatatatataaaggaaagttc
aaatgtgaaccactttttcattgtgaacgtgaggacgaatctcaattactcatccggtcaataaaggcggctttgaccatggtacccctcttta
ataatctctaccctcccatgaatcgtgtcaatcctccgtgattacgtccaccgtaacccaagatgtgtgccacgtggcgcactattggttgtcc
tcacgttcacaatgaaaaaatagttcatatttgaacccacacctatatatatatatatatatatatatatagaggaaggttcaaatgagaagt
gctaaaaaatgcgaagcagagaagtgatttccatcactcatccggtcaataaaggagactttgaccggtccaccactcatacggtggacg
tgatcacggaggattgacgcgatttatgggagggtagagattattaaagggggagaccggtcaaagccgcctttgttgaccgagatga
gtggttgagatcacttctctacttcgtattttttttagcagttctcatttgaacctaatactatatatatatatatatcttggtttccctatgaagacta
cttggatcacaaaatgataggaattactttgttcaacttagttctcttgctaatatgcactattctcctattcatcttcaagctctccacaaaaaac
tctacatcaaattcaaacctacctccaagccctccttcaattcccatacttgggcacctcctcttcttactcaaacaacccctccatgaaaccctacgcaccctctctcaaaaattcggcccaatc
>CtCYP81E14

aagtaaaataagtaaattgtcactagattttcttaacagacactataaaaaagtggttgattatttatgctattttaacaatagtaatacactta
aaaatgttaaaaactataataaagtgtggatatatttggcgttacaattttccatcaaatctctacatttaatagtgaaagttatagtaaaaatc
acgtttattagtgtgaacaattatctaaatttccacatttctaaacatggaaattttgaaaaataattcgaaggttacaaaagtccacgctttaa
agtgtgaacttttctaattacaaaaattacacatttaaaagcgtgaacttttgaaatcaaatgtacacatttataaatatggagtttttttttctaa
caatctttatataaaaaaactatttgtcatacaaactttttccacggcttttaagaaacgtggataaataatatatttttttgaagtgagaaaggg
gaaaacttttataacaccaaccaaacaaagaacaagccaaatacaagcaattaattccaaccacaaaccaccccacaaaacagaaagtat
ttcaaacccacccaaatcaaccaacagacagaaaccaaattaccagctaaaccaaaacacagatacgagcagagactaagtgagcagg
acaggtcgattaacccagaagaactaagaaaacaaaccagtttacgagtagtacccctagatgccgactccctggaccgttctctacggct
aatgcgaccgctgctgtactaagcggagccgctaccagaatagatgcccttcgcgctaccacctgtggttataaactcagtcgtccgatcc
cccgattttagtactccagctagcatgttccattcagccattcgtaccggtccttgtgccgatttgttcttctttagacgacacattcgtggttac
aaaaacccaaacggatcagatagccaagttttaagaaaaccaagttatgatccatttattatcatcgatctaactcaagtaaaacaaactctt
tataatttttctgagatttaagcttgccttgaactcaaacagaggattctgacgggttctgggtgggttaggccgcatccaaaccgccaagc
acactgttgtctgcgacttcagtgaatcaagcgggtatacccgcatccagaccgcccgcttcatttttttagcaccgtttgaagtctgttttcaa
gcttttggttgacaccacatttctcatcttactttttttggtcctctgaatttaattgagaataaaaacgactgtcaaatggctaaaatgcccatca
tatgcctaacggagttaaaatgaacggagttaaaatcagggacccaaagcgttatagaatgaataccatgaggacctaatccataattatt
ttattcgagagatccaaaatattaaaaccatcaaaccacaagtccaaatatgtaattcactcttagttttatatattttagggagtatccattgtt
ctttatttatacactcactcattcatttttatcttctccaaaaataaataaataaataaataaaataactagcttagttgatagctcaagaa
gaagccatagatttctatccattataaaaagctttgtaacatctgggtgtattaccataaagtgataatggatcagttcttgacattttacgtct
atggtttttaccaatttagattttcatataaatatctcgatgtctctcttatcttactttattttgcattttatcgttaaacatggttgcatatccttgat
agttgatcgtgtcatattcatttatcatattttcgttattatctcgctataaaaacgtccatagcccaataatttttaaacttatccaaaacataccc
aaaaaagggcaaaaccccaaaagttataattatttattattacacttatccattaaaaatatcataaccgaaaaaacatgttgtcatggcttgc
tgcataaccacacatttgcca
>CtCYP81E15
caagtcactaatcagcgagctagagtcgctgatccttgggaaggatcagggagtcgctgtgacttgtattcttcagaacataggctagtca
gcttgctgtcactgattccgacttcagatcatgagcatcgctgactctatcattagcttgcatcaatagatgacgctgatggttcttagttgcat
gactgtttgtcttcataaggatacttagcagtgatcagttctagctgccactgaacagactcaggtaggcatgctgtcgctgatgcattatca
gcatgtatgctttgagcagtgaacctatcagcgaacaaactcgctgtagttttcaaagcatcttaattttgctaagtatgtaaagttacttgag
ataggatcattggtatttcatcaggtcataaaggagctggatcaacatgatggcatagaaagattattgccttcgccgtatcctggcaagttt
cgtggtttccatctttgatggttttccaaggcccttggcatctaggtgtatttcggcatccaatacctaagacaagtagtttttttccagtaatgtc
ctaagttaaaaactcgagttttgcaaggtttgacatgattaactataattagatataaaagttaataatggcgaaatttaattttccttagtttta
tactgatttgatatttaggatgtttgaaaagtttaaaaatccataaatagttatttataaatcagcaagtaaataaatcggaaaattgaagtttt
gatctttatttgagaagtcgggattaaaattatgatgactttatattttaagactaggaaaacgcaaccaaagaatcctaagaacgattctat
cattcgtgatagaatcgttagaataagggggtagaaacttattgtttactgaaaaataaattgcgattaaaactgacctctttcaacctctag
atgttcttggatcttcttcgttgttgtttatcttgcaatcgccaatttcaactcctttccaagtgattcgtgctgatagcgtgttataaataatagg
agtagtaggagaggatggtgattggtgagagtgagagagatagagagatgaggatgaaagcttgtaatcttcatatttatagttatagg
tggattagtctccaagaaaaataacatccatccaatacatatatctaaacaataatttgaggatagacgtccattccatataatgatggataa
cacataataagttcatgaaaaaatatcaataaaacagccaacaaataaacaaatgttgatgaaacttcacataatactcgtagatatcacag
gagcaaacttaaaccccaaaccctaaacttatgttatattcagctttaaaccacaatgacatccttctttagatactatagtgtcacaatcaaaa
ttaattaggtactgccctcgggtgagagggttcatcaacaggtttggcgggtggctccccatactggtacgccggctaatgtggaacccg
gagggtgacctcctctcactcatcctttctctctctctctctctccctctctctctctctctctctttctctatctatctctccctctctatcttata
catatatatatatagggaggttcatacgagaaacatgtcaatttaagaaaaagcgaagagcaatctcagccacacatcttgggttacggtg
ggcgtgatcacggaggattgacgcgattcatgggagggtagagattattaaagggaggatcactgtcaaagccgcatttattgaccgga
tgagtggttgagattgcttctcacgttttcttagaaagttatgtttttcatttgaacgtttctctctctctctctatgtatatatatatatatattaa

aataataaaaaaaatccggggttaaagattgagttagttagagtaatggtactccattttaaggtaaggatggtcgaaaagagaaaaagat
aatgtgacattaaggtgacatcaaggttgag
