# Supplementary material for: Molecular Characterization of an Isoflavone 2′-Hydroxylase Gene Revealed Positive Insights into Flavonoid Accumulation and Abiotic Stress Tolerance in Safflower
Source: Molecules. 2022 Nov 18;27(22):8001. doi: 10.3390/molecules27228001 (PMC9697648; doi:10.3390/molecules27228001)
Supplement: Supplementary file 1 [file molecules-27-08001-s001.zip › molecules-2004143-supplementary Table S5.pdf]

Table S5

Primers used for the expression analysis of key flavonoid biosynthetic genes.

Primer code	Primer Sequences (5′-3′)	Use
AtANR1-R<br>AtANR1-F	CGCAATCTCTGGAACACTTGTG<br>AGCTCGTGCCCATTTGTTTC	qRT-PCR assay
AtF3H-R<br>AtF3H-F	ATGTCGAAACGGAGCTTGTC<br>TCAGATCGTTGAGGCTTGTG	qRT-PCR assay
AtDFR1-R<br>AtDFR1-F	GTACCGAACATACATAGTTG<br>CTTCAAGTATAGTCTCGAGG	qRT-PCR assay
AtCHI1-R<br>AtCHI-F	AAGATACTTGGCAATGGTTGCG<br>CGGTATGCAACATGCCGAA	qRT-PCR assay
At4CL-R<br>At4CL-F	ACTCCGTCGTCGTTTTGAAG<br>CGCAAACCCTTTCTTCACTC	qRT-PCR assay
AtF3′H1-R<br>AtF3′H1-F	GACTGAGCTAGCCGGAGAGTC<br>GAGGAGCGTGACCACAA	qRT-PCR assay
AtANS1-R<br>AtANS1-F	TGCCGGTGAAGGAGAAGAAA<br>CAGCCCAATCAAGCTTTTGC	qRT-PCR assay
AtFLS1-R<br>AtFLS1-F	TGAAACAAGTGGTCCACCCAC<br>ACCATAGCCAAGCCTGCAAA	qRT-PCR assay
AtCHS-R<br>AtCHS-F	CAGATGCATGTGACGTTTCC<br>TGAGAACCATGTGCTTCAGG	qRT-PCR assay
At18SrRNA-R<br>At18SrRNA-F	GAAGATGAAGGAGACGAGAA<br>CTACCGTTTCCAAAGGCGAG	qRT-PCR assay
CtCYP81E8-R<br>CtCYP81E8-F	TGCTTTGACTGGGAACGAAC<br>AGAACGAGGCACGCAATTTG	qRT-PCR assay
