# Supplementary material for: Molecular Characterization of an Isoflavone 2′-Hydroxylase Gene Revealed Positive Insights into Flavonoid Accumulation and Abiotic Stress Tolerance in Safflower
Source: Molecules. 2022 Nov 18;27(22):8001. doi: 10.3390/molecules27228001 (PMC9697648; doi:10.3390/molecules27228001)
Supplement: Supplementary file 1 [file molecules-27-08001-s001.zip › molecules-2004143-supplementary Table S6.pdf]

Table S6

WT and OE-13 HPLC-MS/MS analysis test results (μg/g)

Sample	Metabolites	Results	Limit of Quantitation
**WT**	Apigenin	-	0.125
	Catechin	-	0.125
	Epicatechin	Not	0.125
		detected	
	Rutin	Not	0.125
		detected	
	Myricetin	-	0.125
	Dihydromyricetin	-	0.125
	Dihydroquercetin	0.195	0.125
	Kaempfero	-	0.125
	Chalcone	0.28	0.125
	Naringenin	0.695	0.125
**OE-13**	Apigenin	-	0.125
	Catechin	-	0.125
	Epicatechin	Not	0.125
		detected	0.125
	Rutin	Not	0.125
		detected	0.125
	Myricetin	-	0.125
	Dihydromyricetin	-	0.125
	Dihydroquercetin	-	0.125
	Kaempfero	0.205	0.125
	Chalcone	0.183	
	Naringenin	0.228	
