# Supplementary material for: Molecular Characterization of an Isoflavone 2′-Hydroxylase Gene Revealed Positive Insights into Flavonoid Accumulation and Abiotic Stress Tolerance in Safflower
Source: Molecules. 2022 Nov 18;27(22):8001. doi: 10.3390/molecules27228001 (PMC9697648; doi:10.3390/molecules27228001)
Supplement: Supplementary file 1 [file molecules-27-08001-s001.zip › molecules-2004143-supplementary TableS3.pdf]

Genomic sequence of CtCYP81E genes identified from safflower genome

>CtCYP81E1

ATGGAGGTTTTTCTTTATCTCTTCATCTCCATTCTACTCCTTCTATCTTCATACCTCTTCAC
CCGTCGCAAAATCCCGAACCTTCCGCCCTCCGTCTTCCCATCCCTCCCGCTAATCGGCCA
CCTCTACCTCCTCAAACCCCCTCTCTACCGCACCTTCGCCAAGATCGCCTCCAAATCCGG
TCCCGTCCTTCTCCTCCGCCTCGGATCCCGCCGCGTCCTCCTCGTCTCCTCCCCTTCCGCC
GCCGAGGAATGCCTCACGACAAACGACGTCGTCTTCGCCAACCGCCCCAGGATGCTCTT
CGGCAAGATCATCGGCATGAACTACACCAGCCTCGCCTGGTCCCCCTACGGCGACAAC
TGGCGCAACCTCCGCCGCATCGCCGCCACCGAGATCCTCTCCATCCACCGCCTCAACGA
GTTCCACGACGTCCGCGTCGACGAGTCCAGGCTCCTCGTCCGCAAGCTCATGATTCCAT
TCTCCATTCCTTCTAATTCATATTCTAGTTCGAGTTCTTCTTGTTCACATGTGAACATGAA
GTCGGCTTTCTATGAGTTCACGCTGAACGTGATGATGAGGATGATCTCCGGGAAGAGGT
ACTTCGGCGGAGATATTCCGGCGGTGGAGGAGGAAGGGAGGCGGTTCAGGGAGATCTT
GGACGAGACGTTTCTGCTCGCCGGAGCGGCGAACGTCGGCGATTACTTGCCGGTTTTGG
GGTGGTTGGGGGTCAAGGGGTTGGAGAAGAAGTTGATCCGTTTGCAGGAAAAGAGGGA
TGTGTTCTTTCAGGGGCTGATTGAGCAACTTAGGAAATCTAAAGGGGGTGAAAGTGAA
AATAAGAGTAAAAGGAAGACGATGATTGAAGTGTTGTTATCTTTACAAGAATCAGATC
CTGAATACTACACTGATGCTATGATCAGAAGCTTTGTGCTGGTGCTCTTATCTGCCGGCA
CCGATACTTCGGCCGGGACGATGGAATGGGCCATGACTCTTTTGCTAAACAACCCACAT
GTTCTCCGAAAGGCACAAAACGAGATCGACAAAAAAATCGGCAACGATCGTCTCGTTG
ACGAGTCCGACATCGCTGATCTACCTTACCTCCGTGGCATCATAAACGAGACCCTCCGG
TTGTACCCTCCGGGCCCGTTACTAGTCCCCCACGAGTCATCGGACGATTGTGTTGTCGCG
GGCTATAACATCCCAAGTGGGACCATGCTTCTCGTGAACCAGTGGGCCATACATCACGA
CCCGAACCTCTGGACCGAGCCCGAGAGGTTTAATCCGGAAAGGTTTGACGGGCTAGAA
GGGACACGAGACGGGTTTAAGCTGATGCCGTTCGGTTCCGGAAGGAGGAGTTGTCCGG
GGGAAGGGTTGGCGGTTCGTGTGCTTGGTTCAACCCTCGGGTTGCTAATCCAATGCTTCG
ATTGGGAAAGGACGAGTGACAAAATGATTGATATGACCGAAGCTCCTGGGCTTACCAT
GCCCAAGGCTGAACCGTTGGTAGCCAAGTGCCAACCACGGCTAGAGATGCAGAATCTG
CTTTCACAGTTGTGA

>CtCYP81E2

ATGGAGATTTCCTATCTCTACATCTCCTTCCTTCTACTTCTAGCTTCTTATCTTTTCACATT
CCACTTCCGCCGGAGAATCTCCAACCTACCGCCGACCGTCTTCCCGTCCCTCCCCTTAAT
CGGCCATCTTTATCTTCTGAAACCACCCCTCTACCGAACCTTGGCCAAACTCTCCAGCA
AGCACGGCCCCATCCTCCACCTCCGCTTCGGATCCCGCCGTGTCATCTTGGTCTCGTCTC
CTTCTCTCGTCGAAGAATGCCTCATGAAGAACGACATCGTCTTTGCCAACCGCCCACGG

ATGCTATTCGGCAAGATCATCGGCCAGAACTACACGACTTTGGTTTGGTCGCCATACGG
CGACAACTGGCGCAACCTCCGCCGCATAGCATCGATCGAGATTCTATCCATCCATCGCC
TCAATGAGTTCCACGATATACGCGTTGAAGAAGGCAGGCTTCTGATACGTAAACTGCTA
TCGAATTCTTCTCCGGTGAACCTGAAGTTGGTGTTCTATGAGCTCACGCTGAACGTGATG
ATGAGGATGATCTCCGGGAAGAGGTATTTCGGTGGTGATAAATTGGAGGAAGAAGGGA
AACAATTCAGGGCGATATTGAACGATGCGTTTATGCTCGCGGGTGCCGCAAACATGGG
CGATTACTTGCCGATTTTGAGTTGGTTGAGAGTGAACGGATTGGAGAAGAAGTTAATTG
CTTTGCAGGAAAGAAGGAATGTTTTCTTTCAACGGTTAATCGACCAGCTTAGAGAAGGG
AACGGGAATCAAGCGGGAAATAAGAGGAAGACAATGATTGAATTGTTATTATCGTTAC
AAGAATCTGATCCCGAATACTACACCGATGCTATGATCCGAGATTTTGTTCTGGTTTTAT
TAGCAGCTGGGACTGATACTTCATCTGGAACCATGGAATGGACCATGTCTCTTTTATTAA
ACCACCCACATATTCTAAAAAAGGCACAAAACGAGATCGACCGAAACGTTGGCAAAG
ATCGCCTTGTTGATGAATCAGACATTCCTAATCTACCTTACCTCCGTTGTATCATAAACG
AGACCCTACGACTGTACCCAGCAGGACCGATGCTAATTCCACACGAGTCATCACACGA
TTGTGTGGTCGGAGGCTACAACATCCCACGTGGGACCATGCTTCTCGTGAACCAATGGG
CCATCCATCATGACCCTAAGCTGTGGAGCGAGCCAGAAAGGTTCGATCCAGAAAGGTT
TGAAGGGCTAGAAGGCACACGAGATGGGTTCAAGCTGATGCCATTTGGGTCCGGAAGG
AGGAGTTGTCCAGGGGAAGGGTTGGCGGTCCGTATGGTCGGGGTGACGTTGGGTTCGAT
CATCCAATGCTTTGATTGGGAAAGGATGAGTGACGCCATGGTTGACATGAGTGAAGGTC
CTGGACTCAACTTGCCCAAGGCTGAACCCTTGGTCGCTAAGTGTAAACCGCGGCTAGAA
GTGCAGAATCTACTGTCACAGCTAATGTGA

>CtCYP81E3

ATGGAGATTTCCTATCTCTACATCTCCTTCCTACTACTTCTAGCTTCTTATCTTTTCACATC
CCACTTCCGCCGGAGAATCTCCAACCTACCGCCCACCATCTTCCCGTCCTTCCCGATAAT
CGGCCATCTTTATCTTCTGAAACCGCCCCTCTACAGAACCTTGGCCAAACTCTCCCGCA
AGCACGGCCCGATCCTCCACCTCCGCTTCGGATCACGCCGTGTCGTCTTGGTCTCGTCTC
CTTCCGCCGTCGAAGAATGCTTCACGAAGAACGACATCGTCTTTGCCAACCGCCCACGG
ATGCTATTCGGCAAGATCATCGGCCTCAACTACACCACTTTGGCCTGGTCACCGTATGG
CGACAACTGGCGCAACCTCCGCCGCATAGCATCGATCGAGATTTTATCCATCCATCGCC
TGAACGAGTTCCATGATATACGCGTTGAAGAAGGCAGGCTTATGATACGTAAACTGTTA
TCCGGTTCTGATCCGGTGAACGTGAAGTTGGTGTTCTATGAGCTGACGCTGAACGTGAT
GATGAGGATGATATCCGGGAAGAGGTATTTCAGTGGCGAAAAATTGGAGGAAGAAGG
GAAAGAATTCAGAGAGATATTGAACGACACTTTTATTCTCGTGGGTGCTTCAAATATTG
GCGATTACTTGCCGATTTTGAGTTGGTTGGGAGTCAATCGATTGGAGAAAAAGTTAATC
GCATTGCAGGAACGGAGGGATGTTTTCTTCCAACGGTTAATCGACCAGCTTAGAAAAG
GGAACGGGAATCAAGCGGGAAATAAGAGGAAGACGATGATTGAAGTGTTATTATCGTT

ACAAGAATCTGATCCCGAATACTACACTGATGCAATGATCCGAAACTTTGTGCTGGTTT
TATTAGCAGGCGGGACTGATACCTCATCTGGAACCATGGAATGGACCATGTCACTTTTA
CTAAACCACCCACAGGTTCTGGAAAAGGCACAAAATGAGATCGACCGAAATGTTGGCA
AAGACCGTCTCGTTGATGAATCAGACATACCTAATCTACCTTACCTCCGTTGTATCATAA
ACGAGACCCTGCGATTGTACCCACCAGGCCCGATACTAATTCCACACGAGTCATCCGA
CGATTGTGTAGTTGGAGGCTATAACGTCCCCCGTGGGACCATGCTTCTCGTGAACCAGT
GGGCCATCCATCATGACCCGAAGCTGTGGAGCGAGCCAGAAAGGTTCAATCCAGAAAG
GTTTGAAGGGCTAGAAGGCACACGAGATGGGTTCAAACTGATGCCATTTGGGTCCGGA
AGGAGGAGTTGTCCGGGGGAAGGGTTGGCGGTTCGTATGGTTGGGGTGACATTGGGTTC
GATCATCCAATGCTTTGATTGGGAAAGGATGAGTGAAGCCATGGTTGACATGAGCGAA
GGTCCTGGACTCACCTTGCCCAAGGCTGAACCCTTGGTCGCCAAGTGCAAACCGCGGCT
GGAAATGCAGAATCTACTGTCACAACTAACGTGA

>CtCYP81E4

ATGACGAACGGGATGATGGAATCAATTTCGTTGTTGGACTATGCGACCCGTTCATTCAG
GATGACGACCGGAGAGCCAGATTTGGGGTATTTTATGCCAATATTGAAATTGCTGGGAC
TGAATGGTTTGGAGCAAAGGAGCAAGCAGTTGCAGAAAAAAGGAGATTTGCTCATGGA
CAACTTAATTGAAGATCTAAGAACAACAATGCTTGAATTTTCTGATGGATCAGGTGATC
GGAGGGAAGAAAAAGTGCTCGAGTTCTTGCTGGCTAGACAAAAAGACGATCCTAAGCG
TTACCCTGACGAAATTATCAGAGGACTTGTGCTCGTGTTAATGGCATCAGGTACCGACA
CATCTGCCGGAACCATGGAGTGGGCATTTTCTCTTCTATTGAACCATCCAGAAGCTCTAC
AAAAAGCACAGAACGAGATCAAGAATTACGTCGGCAGTGATCGTTTCCTAGAGCAGTC
AGACATTGAGCATCTGCCTTATCTCAATTGCATTGTAAAGGAGACGATGCGAATGTACC
CGGTTGCCCCTTTTCTAGTCCCTCATGAGTCATCAAAGGAGTGTAAAATCGGAGGGTAT
AACGTGCCAAAAGGCACCATGTTAATGGTGAACGTTTGGGCTGTTCAAAGCGATCCCA
ATATATGGGCCGAACCCACAAAGTTTAAGCCAGAAAGGTTCGAAAAGGTTGTAGGCGA
AAGAGATGGATTTAAGCTAATGTCGTTCGGGTATGGAAGGAGGAGTTGTCCTGGGAAG
CACATGGCGGTTCGTGTGATCAGTTTGGCATTGGGCTCGCTTATTCATTTCTTCGACTGG
GAAAGGGTTAGTGAGAAGACCGTTGATCTGACCGAAGAAACCGGACTTGCCTTGCTCA
AGGCACAACCGTTGGTGGCTATTGGACGAGTTTTTGAGGAAAGTGCGGTGAAACCGAA
GAATGGATCAAAGGAGGACGTGGGAGAAGCATGGACGAGCATTGAAGATGGCGGAAA
GTTGAAGAAATCGGAGCTTTTCTGA

>CtCYP81E5

ATGGACAAAGTTTATCTGTTCTTCATTTCCCTCTTATCCTTGCTCTCTATCATCTTGCTTTT
CAAGCCCTTTACACCCAGAAAAAGAAGAAATCTTGCACCATCTCCACCTTCTCTCCCAG
TAATTGGCCATCTCCATCTAATTGGGGAACCTTTTCATAGACTCCTTCACCATCTTTCTTC

CAAGTATGGTCCAGTTATGTCTCTTCGCTTTGGCTCTCGCCCGGTGCTCGTAGTAACCTCT
CCATCGGCCGTCGAAGAGTGCTTCACCACACACGACATCGTCTTAGCCAACCGGCCGCT
CCTTTTAAGCGGCGAGTATCTCGACTACGACCACACCACGATGGGTTCCGCACCTTACG
GACGTCTCTGGAGAGACCTTCGCCGTATCGCCACGCTCGAGCTCTTCTCCGCGGCCCGG
CTCAAGGCCTACATGGGGGTCCGGCACGACGAGGCGCGATCGTTGGTGAAGTCTCTTTC
TCAAGACGTGTCCCACGATTTCACGCGGGTAGAAATGAAGTCGAGAATCGAAGGTTTA
TCGTACAACATAATTATGAGGATCGTTGCGGACAAACGGTTTTATGGGACGGAGGTGG
ACGGCTTTGAGGAAGCTCGGAAGTTTAAGGATCTGATAAGGGAAATCTTTGAGGTGAG
CGGAGCTTCGAATGCCGGAGACTTCATTCCGTTCTTGCGATGGATGGATTTTCAGGGTTT
GGAGAAGAAGCTTCGGAAACTGCAAACCGAATCCGATAGCTTTATACAGAGGCTGATT
GAAGAACGTAGGAGTAAACGCGATAGTACTTCTTCGTGCGATGGAAAGGCCAAGACGT
TTATCGACGCAATGCTTTCTTTGCAGGAATCGGAACCCGAATACTACACCGACGATATA
ATTAAAGGCAATATGTTGACAATGCTAATTGCGGGAACCGACACTTCATCCGTGACGAT
AGAGTGGGGGATGTCACTTCTTCTAAATCATCCAGACGTGCTAAATAGAGCAAGAGCA
GAGATCGATGAACATATTGGACACGAACGTTTGGTAGAGGAGACCGATCTTCCCAATCT
TCCCTACATCCAATGCATCGTCCACGAAACCCTAAGACTATTCCCAGCAGCACCACTAT
TGGTGCCACACGAACCATCGGAAGATTGCACGATCGGGGGTTTCCACGTGGCACGTGG
CACGATCGTGTTGGTGAATGCATGGGCCATCCATAGGGATCCACAGTTATGGGATGAGC
CATTAAGTTTCAAGCCAGAGAGGTTTGAGGAGATTGGAAACATGGGGTATCGGTTCGTA
CCGTTTGGCATGGGCCGTCGACAGTGTCCCGGGGCCGGGTTCGCGAACAGGGTCGTGGC
AACGGTGCTGGCGTCGTTGATCCAATGCTTCGAATGGAAAAGGGTCGGTGACGAGCTTG
TCGATTTGTCGGAAGGCAAAGGTCTAACGATGCCCAAAGAAAAGCCTCTCGAAGCAAT
GTGCAAAGTTCGCCAACGAATGAGTCGGGTTCTTTCCGGGTTATGA

>CtCYP81E6

ATGGTCAATTATCAAGAGTCCACACATTGCAAGCTGATTCTGGTCATGGATATCATGGA
TGAAATTCATCTTCTCTTGGTATCTCTTTTATCCTTCTTCTCTGTGATCATTTTGCTTTCCA
AATTTCTTATACCCAGAAAGACAAGAAATCTTGCACCATCTCCACCTTCTCTCCCAATA
CTTGGCCATCTCCATCTTATTGAAGAACCTTTTCATAGAGTCCTTCACCATCTTTCTTCCA
AATATGGTCCAGTTATGTCCCTTCGCTTTGGCTCTCGCCCAGTGCTCGTAGTAACCTCTC
CATCGGCCGTCGAAGAGTGCTTCACCACAAACGACATCGTCTTAGCCAACCGGCCGCTC
CTTTTAAGCGGCAAGTATATCGGCTACGACCACACCGGGATGGGTGTTGCACCTTACGG
ACGTCTCTGGAGAGACCTTCGCCGTATCGCCGCGCTCGAGCTCTTCTCCGCGGCCCGAC
TCAAGGGCTACGTGGCGGTCCGACAAGACGAAGTGCGATCGTTAGTGAAGTCTCTTTCG
CAGGACGCGTGTCGAGATTTCACGCGGGTGGAAATGAAGGCCAGGATCCAAGGTTTGT
CGTTCAACGTAATCACGAGGATGGTTGCGGACAAACGGTTTTATGGGGCCGAGGTGGA
GGACTTTCCGGAGGCTCGAAAGTTTAAGGACATGATGACGGAAGTGTTCGAAATTAGC

GGAGCATCGAATCCCGGAGATTTCATCCCGTTTTCGCGATGGATTGATTTTCAGGGTTTG
GAGAAGAAGTTTCAGAAATTGCAAGCTAAATTCGATAGCTTCTCCGAAACCCTAATAG
AAGAACGAAGAAGGAGTAAGCGCGATGATTCTTCGGGCGAATGGAAAGCGAAGACAT
ATATCGATGCTTTGCTTTCTTTGCAAGAATCACAACCAGAAGAATATACCGACAATATA
ATCAAAGGCAATATACTGACATTGCTACTCGCGGGAACCGACACTTCATCAGTGACGAT
AGAGTGGGGGATGTCACTTCTTTTAAATCATCCCGAAGCGCTAAAGAGAGCTAGAGCA
GAGATCGATGAACATATTGGACATGAACGTTTGGTGGAAGAGACCGATCTCCCCGATCT
ACCATACATGCAATGCATTGTCCAAGAAACCCTAAGACTATTCCCAGCAGGACCGTTGT
TGGTGCCACACGAATCATCTGAAGATTGCACGATCGGAGGGTTTCACGTGTCACGAGGC
ACAATGGTGTTGGTGAACGCGTGGGCCATCCATAGGGATCCAGAGTTATGGGACGAGC
CATTAAGTTTCAAGCCCGAGAGGTTTGAGAAAACGGAAAACATGGGGTATCGGTTTAT
ACCGTTTGGAACGGGACGTCGCCATTGTCCTGGGACGGGGCTTGCGAACAGGGTCGTG
ACGTCGGCGTTGGCGTCGTTGATCCAATGCTTCGAGTGGGAAAGGGTTGGTGAAGAGGT
GGTGGAGTTGTTAGAAGGCAAAGGTCTATCCATGCCCAAAAAGAAGCCTTTAGAGGCA
ATGTGTAAAGCTAGACAAAGAATGAGTCATGTTCTTAAGGAGCTATAA

>CtCYP81E7

ATGTCCCTTCGCTTTGGCTCTCGCCAAATACTCGTAGTAACCTCTCCGTCGGCCGTCGAA
GAGTGCTTCACCACAAACGACATCGTCTTAGCCAATCGCCCGCTCCTTTTAAGCGGCAA
GTATCTCGACTACGACCACACCACGATGGGCGCCGCACCTTACGGGCGTCTCTGGAGA
GACCTTCGCCGTGTCGCCACGCTCGAGCTCTTCTCCGCGGCCCGACTCAAGGGCTACAT
GGCGGTCCGACAAGACGAAGTGCGATCGTTGGTGAGGTCTCTTCTTTCTCACGACGCGT
GCCGAGGTTTCACCCGAGTGGAAGTGAGGTCCAGGATCCAAGGGTTGTCGTTCAACGTG
ATCATGAGGATCGTTGCCGACAAACGGTTTTACGGGGCCGGGGTGGACGACGTTCAGG
AGGCTCGTAGGTTTAAGGACGTAATGAGGGAAGTGTTTGAAATTAGCGTAGCGTCGAAT
CCCGGAGATTTCATCCCGTTTTTGCGATGGGTTGATTTTCAGGGTTTGGAGAAGAAGCTT
CAGAAATTACAAGCGAAATTCGATAGCTTTTCCGAAGCCTTAATAGAAGAACGTAGGA
GTAAACGCGAGGGTTCTTCGGGAGAATCGGAGGCCAAGACATATATCGATGCTTTGCTT
TCTTTGCAGGAATCAGAACCGGAACATTATACCGACAATATAATCAAAGGCAATATAC
TGACATTGCTACTCGCGGGAACCGACACTTCATCAGTGACGATAGAGTGGGCGATGTCA
CTTCTTCTAAATCATCCCGAAGTGCTAAAGAGAGCCAGAGCAGAGATCGATGAACATA
TTGGACATGAACGTTTGGTGGAAGAGACCGATCTTCCCAATCTACCATACATCCAATGC
ATTGTCCACGAAACCCTAAGACTATTCCCAGCAGCACCGTTGTTGGTGCCACACGAATC
ATCTGAAGATTGCACGATCGGAGGGTTTCACGTGTCACGAGGCACAATGGTGTTGGTGA
ACGCGTGGGCCATCCATAGGGACCCACAGTTATGGGACGAGCCATTAAGTTTCAAGCC
CGAGAGGTTTGAGAAAACGGAAAACATGGGGTATCGGTTTATACCGTTTGGAACGGGA
CGTCGCCAGTGTCCTGGGTCGGGGCTCGCGAACAGGGTCGTGACGTCGGCGTTGGCGTC

GTTGATCCAATGCTTCGAGTGGGAAAGGGTTGGTGAAGAGATGGTGGAGTTGTTAGAA
GGCAAAGGTCTATCCATGCCCAAAAAGAAGCCTTTAGAGGCAATGTGTAAAGCTAGAC
AAAGAATGAGTCATCTTCTTAAGGAGCTATAA

>CtCYP81E8

ATGATGAGGATGATTAGTGGTAAGAGATATTATTATGACGTCGATGATGTACTGATCAC
CGTGGAAGAGAAGGAAAAGGCAGATTGGTTTCAGGAACTTGTGACGGAGATTTTCGAG
GTTATGGGGGCTACAAACGTAGGGGACTACTTGCCGATGATGAGGTGGTTGGGAGTGA
GCAAATTGGAGAAGCGAATGATTCGTTTGCAGGCAAAGCGAGACTTGTTCATGCAGGA
GCTAGTGGAGGAGCTGAAAAGAGGGCTGAATAAGAGTACTGATGATCAAGAGAAGAA
GAAGAAGAAGAAGAAGAGGAGGAATAACATGATTGATATGTTATTATCTCTGCAAAAA
ACCGAACCCGAGTTCTACACCGATGAAATGATCCGAAGCATCATGCTGGTGATGTTAGC
GGGGGGCACTCAAACGTCCATATCTACCATGGAGTGGACAATGTCTCTTTTACTCAAAA
ATCCAAGTGTTCTAAAAAAGGCACAGGCTGAGATCGATAAATACGTGGGCCCAGATCA
TCTTATCCAGGAATCCGACATTCCTAACCTGCCGTATCTTGGTTGCATAATAAAAGAAA
CCATGAGGATGTTTCCGGCAGGTCCTTTGTTGCCTCATGAGTCCACGAAAGATTGCAAG
GTCGGAGGATATCACATCCCTGGTGGCACGATGTTATTAGTGAACGTGTGGGCGATACA
AAATGATCCAACCATATGGGGCGACCCAAAAACGTTTAGACCCGAGAGGTTTGAAGGT
CTAGAAGGTTATACAGACGGTTTCAAGTTTATGCCATTTGGGTTCGGAAGGAGAAGTTG
TCCTGGGGAAGGCATGGCGAAGCGGATGGTTGCGTTGGGGCTGGGATCGCTTATCCAGT
GCTTTGACTGGGAACGAACAAGTGAAAGCGAGGTTGATTTGAGTGAAGGAACGGGAGT
TAGCATGCCGAAGGCTATAAATTTGGTGGCAAATTGCGTGCCTCGTTCTACAATGTTAA
ATCTATTATCGCATCTTTGA

>CtCYP81E9

ATGTCATCGGACGGCAACATCGCCGACGCCAGTGGTCGTCGGAGGTGGTCTTCGACGGT
CGTCACCGACGATGGTGGTCGACGGCGGTCGGTGGAGGATTTGATAGTTGGTGGAACTG
ATACATCTACAATAACCATGGAATGGGCAATGTCCGAGCTTATTAAGCAACCCTATCAA
ATAGAGAAGGCAAGGGAAGAGCTCGATAGGGTGATTGATTGCAAAATGGCTGGATATG
ACATCACCAAGGGGACAATAGCACTCGTTAATGCATGGAGCATCGGGCATGACTCAGA
TCATTGGGATGATCCAGAGGATTTTCGACCCGAGCGATTTTTGGGAAGGATATAG

>CtCYP81E10

ACAAGTTCCGTAAATTCCCCACCACGACCGTTTTTCTCCTTACCCATAATCGGCCACCTC
TATCTCTTGAACCAACCGCTGCATCGAACCCTTGCCAAGATTTCGAACTGCTACGGTCC
GGTCGTGTTTCTGGAGCTCGGTTCGCGCCCGGTGCTCCTGGTCTCCTCTCCGGCTGCAGC
CGAGGACTGCTTCACCACAAACGACCTAACCTTCGCCAACCGCCCAAAGTTGCTCGTCG

GAAAACACCTCGGATATAACTACACAACACTCACTTGGGCCTCATACGGACCCCACTG
GCGAAACTTACGACGAATTGCTACCCTCGAGATTTTATCGGCCAATCGTATCCAGAAGT
TCACAGGTATTCGTCGTGATGAGATAATCTCCCTAGTTTCTAACTTATTTGCGGGCTCTC
GGAAGGATCGATTTGTGGTTGCAGATATGAAGTCGTGCTTTTTTAAGGTGACGTTGAAT
ACGATCATGATGATGATCTCTGGGAGGAGATGTTACGGTGAGGATATGGAAGGACCGG
TGGCCGGAAAGTTGCAGGAGATGGTGGAGGAGACGTTCCGGTTGAGTGGTAGTACGTC
GAATATCGGAGATTTTTTGCCGTTGGCGAAGTGGGTCGGGTTGAATTCGATGGAGAAGA
AGATGGAAGAGCTGAATTTGAAGAAGGATTCAGCCATGCAAGAGATGATTGAATGGCA
TAGGAGGATGAGGAGTGATTCAGCTTGTTTATGA

>CtCYP81E11

ATGGCGATCAATGAACTCGAAAACAAAGTGGGCTCGTCAAGACTTATGAATGATTCAG
ATCTTTCCAATCTTCCCTATCTTCATGGAGTTATAAACGAGACATTTCGAATGTGCCCGG
CTGCACCTCTAATGGCACCCCACGAATCCACAAAAGAATGCTTTGTGAGCGGTTTTCGG
GTTCCAGCCGGAACAATGCTTTTGGTGAACCTTTGGGCCATTCAAAACGATCCCAAGTT
ATGGAAGGAGGCTGAAAGATTTAAGCCAGAAAGGTGGATTGAGGTTGAAGGGCAGAG
AGATGGGTTTAAGTTTATGCCATTTGGGTCGGGTCGAAGAGGGTGCCCTGGAGAAGGGT
TGGCGATGCGAATGGTTGGATTAAGTCTAGGGATGTTGCTTCAATGTTTTGAGTGGGAG
AGAATTGGTGTGGAGAAGGTGGATATGAGTGAAGGGTTTGGTCTCACAATGCCGAAAG
CAAGCCCTTTGGTTGCTAAATATCAGCCACGACCGCATATGGTGGATCTTCTTTCTCGCA
TATAG

>CtCYP81E12

ATGTTACTCCGTTTCGGTGCTCGAAGAGTACTAGTCGTCACCTCTCCTTCGGCCGTCGAA
GAATGTTTTACCAAAGGCAATGACATCGTTTTCGCCAATAGACCTTTTGCGGCATTCGGT
AAATACTTGAATTATGATAACACCACCATGGGCTTCGCCCCATACGGCCACCATTGGCG
CGCCCTCCGCCGAGTTGCCGCCTCCGAGCTTCTCTCCACCGCCCATCTCGACTCAACCA
CCACTTCACGTGAACACGAAGTGAAACTGTGCTGTCGGGAAATCGTTAGAGAGTCGTTG
TCATCACAAAAAATTGAGCTTCGGTCGAGGTTTAGCGGCCTTGTGAATAACATAACGAC
AATGGCGATGATGGGGAAGAGGTATTATGGAGATGGGGTCGAAAAGGGCGATGAGCG
ATCGGCCGCAATATATCGGGAGATTATGCGGGAGATGTTCAAGTTCATACATACACAA
AATCTTGGAGATTTCTTGCCGATTTTAGGGTGGGTTGATTTTGGGGGTACCAAAAAGAA
GATGGTTAGTTTAATGAAGAGAGTAGATGAGTTCTTGGATGAGGTTATAGAGGTACGAC
GTCGTATTGGAAGATGTGGATCAGGGATTATGGTTGATAAGCTGTTGGAATTGCAAGAG
GAGGTACCAGAGATTTATACTGATCAATTCATCAAAGGCCTCATACTGATAATGCTAGT
TGCGGGTACAGAAACACCATATTCAACACTCGAGTGGGCGATGTCTTTGTTGCTCAACA
ATCCAAAAGCCATTGAGAAAATCAAGAACGAGATCGATACTCACATTGGATCTAATCG

TCTATTACAAGAAAACGATTTGGAGAAACTAAGTTACCTTCGAAACTTCATCATTGAGA
CTCTACGTTTGTATCCAACATTACCCTTGTTACTCCCTCACAGTGCCTCGAAAGATTGTA
AAATAGGCGGCTATTTGGTTCCTGGTGGAACAATGTTGATAGTGAATGCATGGGCCATC
CACAGGGACCCCATCTTGTGGGATAAACCAGAAGAATTTATACCGGAAAGGATTGAAG
GGACATGTGATCAGGATATCAAATATAAGATGTTGCCATTTGGCGTTGGTAGGCGTGGA
TGTCCAGGAAACAGATTAGGGTTTAGGGTGTTAGGGTTGACGTTAGGAACTTTGATTCA
AGCATTCGAATGGGAGAAGATTGGTAATGAGGATGTTGACATGACTGCTTTTTATGGCA
CTTCCATGTCCAAATTGAAGCCACTGCAAGCTCTTTGCAAACCTCGTTCAATGATGGCTC
ATTTTGTCTAA

>CtCYP81E13

ATGTTGCTCCGTTTCGGTGTCCAAAGAGTACTAGTCGTCACCTCTCCTTCTGCCGTCGAA
GAATGCTTTACCAAAGGTAACGATATCGTTTTTGCCAATAGATCCCTTTCGGCCCTTGGA
AAATACCTCAATTACGATTACACTACCATGGGCTTCGCCCCATACGGCCACCATTGGCG
CGCCCTCCGCCGAGTTGCCGCCTCTGAGCTGTTCTCCACCGCCCATCTCGACTCAACCAC
CCGTTCACGTGAACACGAAGTGAAACTGTGCTGTCGGGAAATCGTTAGAGAGTCATTGT
CATCACAAAAAATTGAGCTTCGGTCGAGGTTTAGCGGCCTTTTGAATAACATAACGACA
ATGGCGATGATGGGAAAGAGGTATTATGGAGATGGGATCGAAAAGGGCGATGAGCGA
TCAGCCGTGATATATCGGGAGATTATGGGGGAGATGTTCAAGTTCATACATACACAAA
ATCTTGGAGATTTCTTGCCGATTTTAGGGTGGGTTGATTTTGGGGGTACCAAAAAGAAG
ATGGTTAGTTTAATGAAGAGAGTAGATGAGTTCTTGGATGAGGTTATAGAGGTACGACG
TCGTGTTGGAAGATGTGGATCGGGGATTATGGTTGATAAGCTGTTGGAATTGCAAGAGG
AAGTACCAGAGGTTTATACTCATCAATTCATAAAAGGCCTCATCCTGGTAATGTTGGTT
ACGGGTACAGAAACACCATATTCAACAATCGAGTGGGCAATGTCTTTGTTGCTCAACAA
TCCAAAAGCCATGAAGAAAATCAAGAACGAGATCGATACTCACGTTGGATCTAATCGT
TTATTACAAGAAAATGATTTGGGAAAACTAAGTTACCTTCAAAACTTCATCAATGAGAC
TCTACGTTTGTATCCAATATTACCCTTGTTACTCCCACACAGAGCCTCGAAAGATTGCAA
AATAGGTGGCTATATAGTTCCTAGTGGAACATTGTTGATGGTGAATGCATGGGCCATCC
ACAGGGATCCCATCTTGTGGGATAAACCAGAAGAATTTATGCCAGAAAGGTTTGAAGG
GACATGTGATCAGGATATAAAATATAAGATGTTGCCATTTGGCGTTGGTAGGCGCGGAT
GTCCAGGAATCAACTTAGGGCTTAGGGTGTTAGGGTTGACGTTAGGAACTTTGATTCAA
GCATTCGAATGGGAGAAGATTGGTAATGAGGATGTTGACATGATTGCTTTCTATGGCAC
TTCCATGTCCAAATTGAAGCCACTGCAAGCTCTTTGCAAACCTCGTCCAATAATGGCTC
ATTTTTCTAAGGTTTAA

>CtCYP81E14

ATGGATTATTATCTCTTCCTCACCCTCCTCCTCCTTATCATCCTACTTCCATTTTTCTTAGC
CGCCCACTTTCGCCGGAAAACCCAGAACCTACCGCCGACGATCTTCCCAACCCTCCCCA
TCATCGGCCACCTCCACCTTCTAAAGACACCCATCCACCGAACCCTCGCCGCAATCTCC
GCAGAACACGGCCCAATCCTCCTCATCCGCCGCGTCCTCTTGGTCTCCTCCCCTTCCGCC
GCTGAAGAATGCTTCACCAAGAACGACATCGTCTTCGCCAACCGCCCTCGCTTACTCGC
CGGCAAGATTCTGGGCTTCAACTACACCAGCATCGGCTGGGCACCGTATGGCGACCACT
GGCGGAACCTCCGCCGGATCGCCGCCATCGAGATCTTCTCCTCCCACCGGCTCAACGAC
TTCCACGACGCACGGGCCGACGAAGGAAGACTGCTGATACAGAAGCTGGTCTCGGATT
GCTCGTCGCCAGCAAGGGCAAATCTATGTAGAGACCAGATTCACCACTGGTCGCCGGT
GAACTTGAAGTCGGTATTTCAGGAGTTGACGTTGAACGTGATGATGAGGATGATTTCCG
GGAAGAGATACTTTAACGAGGAGATGGATGATGAGGAAGGGAGACGGTTTCGGGAGA
TAGTTAAGGAGACGTTTGTGGTGGGTGGTGCTTCGAATTTGGTGGATCACTTGCCGGTTT
TGAGTTGGTTGGGAGTGAAACGTTTTGAGAAGAAGCTGGTTTCGTTGCAGAAAAAGAG
AGATTCGTTCGTTCAAGATTTGATCGATCAATTTAGAAACGCAAAACCGAAAGATATCA
ACGACAACAAGAAGAAGACGATGATTGAAGTGTTGTTGATGCTTCAAGAAACAGATCC
TGATTACTACACAGATGAACTTATTAAAGCTTTTGTGCTGAACCTACTAACAGCTGGAA
CCGACACTTCTTCTACAACTATGGAATGGGCTTTTTCCCTTTTGCTCAACCATCCAGACG
TTCTAAAGAAAGCACAAAACGAAATCGACACCCACATGATGAACCACAACCAGATCA
ACCGTCTTGTTGACGAATCAGACATGGCCAGCCTACCTTACCTCCGATGCATTCTAAAC
GAGACCTTGCGAATGTACCCAGCCGGCCCGCTGCTAGTTCCTCACGAGTCATCAGAAG
ATTGCGTGGTGGGAGGCTATCACATCCCACGTGGCACCATGCTACTCGTCAATCAATGG
GCCATCCATCATGATCCCGCCTTGTGGAGTGAGCCCGGAAGGTTCGACCCGGAAAGGTT
CCAAGGGGTGGAAGGCACGAGAGATGGGTTTAGGTTTATGGCATTTGGGTTCGGAAGG
AGGAGTTGCCCAGGGGAAGGGTTGGCGATGCGTATGGTTGGGTTGACGTTGGGGTTGCT
GATCCAATGCTTCGATTGGGAAAGGGTTAGTGAAGAGATGGTTGATATGAGCGAAGGT
GCCGGCCTCACCATGCCCAAGGCCCAACCGTGCGTAGCCAAATGTAGACCGCGTCCAA
TGATGCACAAGTTGATATCCTAG

>CtCYP81E15

ATGACAATTTTCACCCTTCAATTTGGGTCATACCCAGTTCTTATTCTATCTTCACCATCCA
CCGTCGAAGAATGCTTCACAAAAAACGATGTTTCCGAAGACCGGCCACGTTTGATCAC
CAGAAAGATCATGAAATATGACTACACCTCCATTATCGCGGCCCCTTTTGGCCCATATT
GGCGGAAGCTCCGCCGTATCACCACCATTGAACTATTCTCGATTACACGTCTCAGCACC
TATCTGGATATCAGACAGGATGAAATTCGGTCGTTGATCAAAACACTATTTCGGGAATC
TGTGGATCATGATTATTTCACGAAAGTGGGATTGAGATCAAAACTTCAAGATATGTCCT
TTAATATCATTATGAGGATTGTTTCCGGGAAACGAAACTTTGGACCTGAACTCAATGAT

TTAAAGGAAGAGATTAACTTTAGGGATATGATCACGGAGATCTTGAAAGTTGGTAGGG
GTTCCTATCGATCAGATCTTTTTCCATTCTTGCGGTGGATCGATTTCCAGGGTATGAAGA
TGACATTATCGAGAATAAAGGCGAAACGTGATGCATTCTTGGATGAAAATGGGGTAAA
GGATGGGGTTTCTAACAATAAAATGATTGATGCCATGCTCTCTTTGCATGAATCTAAGC
CTGAAAGTTATTCGGATCAGATACTCAAGGGAATTATATTGACAATATTACTTGTGTGT
ACGGATTCTTCTGCGGTTACAATAGAATGGGCTATGTCACTTCTCCTAAACCATCCACA
CGTTTTACAAAAAGCTAGAACCGAGGTTGATGAGCATGTAGGACAAGGCCACATGATA
CAGGAACACGAGCTTCATAAACTACGATACCTACAAAACATAGTGAATGAGACTTTAA
GATTATTTCCCACAACTCCTCTCTTCGTGCCACATGAGTCCTCAAAAGATTGCACCATAG
GAGGCTATCGCATACCACGTGGCACGATGTTATTGGTGAACACGTGGGCCATCCACTAT
GATCCAAAGGTCTGGGACGACCCCACAAGTTTTAGGCCAGAAAGATTCGAAAAGCCAG
TTGGAGAAGGGTATAATTATATACCATTTGGAATGGGAAAAAGGCAATATCCTGGAGC
TGGGCTTGCAAATAGAGAGGTTGGAATGGCTTTAGCAGCCATGGTTCAATGTTTTGAGT
GGGATAGGGTTACTGAGAAGATGGTTGACTTAAATGAAGGTAAAGGGTTAATCATGCC
AAAAAATGAGCCACTAGAGGCCATGTGTAGAACAAGAGAAAGTATGGTTAATGTGCTC
TCAAAATTATAA
